# The role of future planning, patience, and risk tolerance for prospective reciprocity in human adults

**DOI:** 10.1038/s41598-026-42226-3

**Published:** 2026-03-06

**Authors:** Stefanie Keupp, Sebastian Grüneisen, Sebastian Olschewski, Maria Victoria Hernández-Lloreda, Felix Warneken, Elliot A. Ludvig, Alicia P. Melis

**Affiliations:** 1 Department of Experimental Psychology, University College London, South Parks Road, Oxford, OX1 3EL UK; 2 Department of Psychology, University of Warwick, Coventry, England; 3 Department of Psychology, University of Michigan, 500 S State St, Ann Arbor, MI 48109 USA; 4 Faculty of Education, Leipzig University, Leipzig, Saxony Germany; 5 Department of Psychology, University of Basel, Basel, Switzerland; 6 Department of Psychobiology and Methodology in Behavioral Sciences, Universidad Complutense de Madrid, Barcelona, Spain

**Keywords:** Psychology, Human behaviour

## Abstract

The current study aimed to test a suite of psychological proximate mechanisms thought to underlie prospective reciprocity – engaging in cooperative acts with the anticipation that benefits will be returned in the future. We developed and validated a novel online test battery with 297 participants to assess individual differences in three domains (planning, patience, risk tolerance) and their relation to participants’ responses across different prospective reciprocity tasks. Tasks assessing patience showed high internal consistency and were positively associated with cooperative behaviour across all three prospective reciprocity tasks. The pattern was less clear for risk, with associations varying across tasks, and planning ability was negatively correlated with one prospective reciprocity task. Across the prospective reciprocity tasks, performance showed limited convergence, indicating that forward-looking cooperative behavior is context dependent rather than reflecting a unitary, domain-general tendency. Taken together, our results show that planning, patience, and risk tolerance each contribute to forward-looking cooperation in task-specific ways, underscoring the need for improved psychometric approaches.

**Supplementary Information:**

The online version contains supplementary material available at 10.1038/s41598-026-42226-3.

## Introduction

Direct reciprocity is an important mechanism to sustain cooperation, enabling genetically unrelated individuals to accrue delayed mutual benefits by exchanging favors over time. Such cooperation involves tolerating immediate costs to the agent that can be recouped later when a former beneficiary returns a favour^1^; reciprocity is thus defined by the behavioural contingency between favors given and received. For reciprocity to exist, individuals must adjust their cooperative actions depending on whether a partner has helped them before or whether they might help them again in the future.

In the current study, we investigate the role of three mechanisms that might support direct reciprocity (hereinafter reciprocity): planning, patience, and risk tolerance. Conceptually, reciprocity can be divided into retrospective (also referred to as past-driven) reciprocity, in which agents pay back a received favor, and prospective (also referred to as future-oriented or strategic) reciprocity, in which individuals initiate a cooperative act anticipating that the recipient will reciprocate at a future moment in time^2^(See Box 1). This distinction is important because retrospective reciprocity is widespread across species, whereas prospective reciprocity has so far been empirically documented only in humans^3–5^.

One possible reason why retrospective reciprocity is more common among a wider range of species is related to the psychological mechanisms that are required: Retrospective reciprocity is likely less cognitively demanding than prospective reciprocity. Specifically, retrospective reciprocity requires individuals to keep track of past interactions with others, preferentially cooperating with individuals who were helpful in the past^2,6^. This can be achieved through ‘attitudinal’ reciprocity (sometimes referred to as ‘emotional bookkeeping’) in which individuals act altruistically preferentially towards social partners with whom they have close social bonds and share a positive emotional history. Another form of retrospective reciprocity is ‘calculated’ reciprocity (for an overview see^7^, where individuals keep track of given and received favors. This form of reciprocity has greater memory demands because individuals have to remember the amount of help received by specific individuals. Several primate species engage in retrospective reciprocity that is grounded in attitudinal reciprocity as its mechanism^3^, but there is limited evidence for the existence of calculated reciprocity in nonhuman primates, possibly due to the additional cognitive and behavioral mechanisms required^2^.

To date, prospective reciprocity has only been empirically demonstrated in humans; its presence in other species remains an open empirical question. Prospective reciprocity requires individuals to strategically invest in others in anticipation of future payback, with cooperative acts in the present being motivated by the possibility of future selfish benefits^2,4,5^. The level of intentional strategizing in prospective reciprocity could vary, as individuals could in principle also feel a stronger bond toward others with whom they expect to interact regularly in the future, eliciting prosocial acts based on an evolved or learned heuristic without the need for explicit reasoning about others’ subsequent behaviors^8,9^. At a minimum, however, this mechanism would require the capacity to identify situations that present opportunities for future reciprocation. Humans are capable of distinguishing one-shot from repeated interactions and also of recognizing situations with prospects for future interaction. They behave more cooperatively in repeated settings and when they believe they may interact again, likely in anticipation of possible reciprocation^7,5^. Thus, individual differences in the tendency to think ahead and plan for the future may relate to this kind of future-oriented reciprocity^10^.

Most research to date has focused on whether humans and other species show certain kinds of cooperative behaviors, while comparatively less work has investigated the psychological mechanisms underpinning these behaviors. Yet, knowing more about the psychological mechanisms that play a role for certain types of cooperation is important as it can help to explain individual differences in cooperative tendencies among human adults and contribute to our understanding of the development of cooperation. In particular, developmental studies provide insight into whether different forms of reciprocity are supported by distinct psychological mechanisms. Several studies have found different developmental trajectories for retrospective and prospective reciprocity: Retrospective reciprocity develops starting around 2–3 years of age^7^, while children begin to engage in prospective reciprocity only from around 5 years of age onwards^11–14^. Furthermore, the emergence of prospective reciprocity is related to capacities for prospection and delay of gratification^12^. Taken together, developmental evidence points to an important difference between retrospective and prospective reciprocity, likely reflecting differences in the underlying psychological mechanisms required.

But what are the psychological mechanisms supporting prospective reciprocity in human adults? Answering this question will help uncover (i) the proximate mechanism underlying the human capacity to strategically initiate reciprocal interactions, (ii) why some people are more likely to engage in prospective reciprocity than others, and (iii) why evidence for reciprocity in animals seems to be limited to retrospective reciprocity based on backward-looking mechanisms.

Several theoretical approaches have highlighted planning, patience, and risk tolerance as critical prerequisites for reciprocity^1,2,14,15^. Though our study focuses exclusively on these three mechanisms, other mechanisms are relevant for reciprocity as well. For example, there is evidence that theory of mind influences levels of cooperation in children and human adults^16,17^. Numerical discrimination^15^, the ability to identify and remembers social partners^15,18^, and trust in their social environments^19,20^,see also^21^ are other critical variables that can influence reciprocity. The former two abilities likely play a more important role in retrospective reciprocity, when individuals need to keep track of given and received favors.

Our rationale, however, is that a specific challenge for prospective reciprocity concerns the time delay between the decision to cooperate and the possibilities for reciprocation. For individuals to make a strategic decision to invest in others who may reciprocate later on, future-oriented strategies (e.g. future thinking, planning and patience) may be necessary. Furthermore, recent results from developmental psychology have shown a clear link between the emergence of children’s prospection and delay of gratification and the emergence of prospective reciprocity between three and five years of age^12^.

The aim of the current study is thus to systematically evaluate the relationship between participants’ abilities or propensities to engage in future planning, patience, and risky decision-making to their behavior in prospective-reciprocity tasks.

In prospective reciprocity, possible cooperators need to be able to think ahead and plan their investment decisions. Limitations in the ability or willingness to think and plan for the future would also entail limited ability for prospective reciprocity. Prospection or future-directed thinking has been shown to enhance prosocial behaviour in children^12^ and adult humans^10,22,23^. Recent work^22,23^ found that experimentally inducing future-oriented thinking promotes the intention to help as well as actual helping. There is also experimental evidence^10^ that inducing future-oriented thinking increases reputation-based generosity, and that this effect is mediated by reputational concern and people becoming more attuned to the consequences of their actions^10^. These works provide the first indications that prospection plays a role in generosity acts. Consequently, the rationale in our study is that people who are more likely to anticipate future interactions with others would be more likely to engage in cooperative behavior that has the potential to benefit them in the future in dyadic interactions. We thus expect performance in experimental tasks that measure future planning to be positively related to cooperation in prospective-reciprocity tasks.

Cooperators also need to be willing and able to tolerate delayed gratification until potential future benefits can be reaped, such as reciprocated favors or somebody else taking a turn in forgoing a reward. Limited patience would entail giving in to immediate temptations and preventing investment in reciprocal interactions. Several empirical studies have focused on the role of time preferences on cooperation, and evidence suggesting that future-oriented individuals or individuals with low discounting rates cooperate more is starting to accumulate^24–27^. These previous empirical studies have investigated cooperation in the context of public-goods games and social dilemmas where individuals make simultaneous decisions about whether or not to cooperate to obtain a shared good, but it is unclear whether this relationship holds when individuals need to take turns rather than synchronize cooperation, as is the case with prospective reciprocity. The current focus is therefore on decisions that involve trusting or helping others who may or may not reciprocate in a later interaction. The rationale is that, for such a reciprocal interaction to get off the ground, people have to overcome the temptation to choose an immediate, selfish benefit in anticipation of a larger, delayed benefit that they can receive through reciprocation in the future. We thus expect the choice of delayed reward options to be positively related to cooperation in prospective reciprocity tasks.

Finally, in prospective reciprocity, there is uncertainty about the possible benefits given the risk that others might not reciprocate. Potential cooperators must be willing to tolerate some risk in order to invest in others, hence pronounced risk aversion may limit investment in an uncertain future. Evidence about the relation between participants’ risk attitudes and their behavior in trust games is mixed^28–30^. The Trust game, a game that we also used in our battery of tasks, is an experimental economic game that measures trust and trustworthiness in two players^19^. Whereas some studies did not find a strong relationship between participants’ risk attitudes and their trust decisions^29,30^, others found that among people who sent anything at all in the Trust game, those who made more risky decisions also sent higher amounts^28^. Engaging in prospective reciprocity entails having to weigh the likelihood of a larger payoff via the reciprocal interaction against the risk of no payoff (or even a loss) through non-reciprocation by a partner. Thus, we expect that individual differences in risk taking will be positively related to cooperation in prospective reciprocity tasks.

Despite theoretical reasons and some empirical evidence that these psychological mechanisms are critical for prospective reciprocity, little empirical research has been devoted to this question, and none has examined all these metrics in the same sample. Our goal is to measure variability among participants^26,31,32^ in patience, planning and risk preferences and use this heterogeneity to learn more about the psychological underpinnings of prospective reciprocity. For instance, if planning or patience are indeed related to prospective reciprocity, individuals with less prospection skills or high temporal discounting rates might be less likely to engage in future-oriented helpful acts.

To test these hypotheses, we presented participants with a series of tasks tapping into these three critical psychological domains and relate individuals’ response profiles to their behavior in several reciprocity tasks. The test battery consisted of ten tasks: one planning task, three patience tasks, three risk tasks, and three reciprocity tasks. The reciprocity tasks we included comprise a trust game^19,33^, a centipede game^34–36^ and the Zurich Prosocial Game^37^. These three interactive games presented several commonalities but also some differences with each other. In all three tasks, participants and an anonymous partner made sequential cooperative decisions—rather than simultaneous decisions, as in other cooperative games, such as the Prisoners Dilemma or Public-Goods Games. In addition, in all three tasks, participants always made the first move and had to risk trusting or investing in an uncooperative partner. Importantly, across all three tasks, cooperation was assessed in settings where future reciprocation was possible, either through explicit contrasts between reciprocation-possible and reciprocation-impossible conditions or through the sequential structure of the interaction itself. This design allows us to target forward-looking, strategic cooperation relative to general generosity or unconditional altruism. At the same time, there were differences regarding the time delay between participants’ first move and the possibility for reciprocation (e.g. shorter in the Trust game than in the Zurich Prosocial game), as well as with regards to the currencies of cooperation (money sharing in the Trust game and instrumental helping in the Zurich Prosocial Game).

**Fig. 1:**
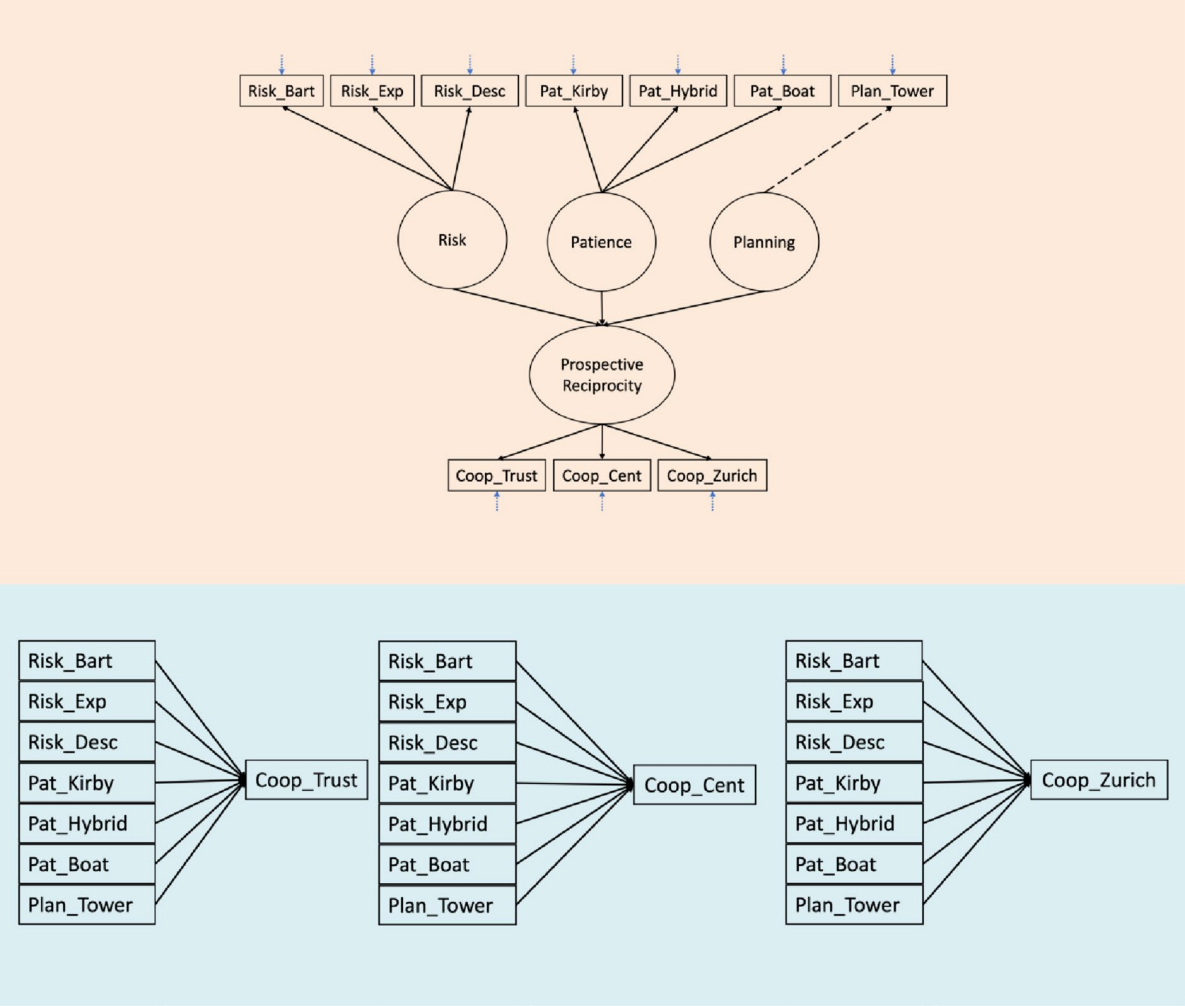
Schematic representation of the two conceptual approaches. Upper panel shows the structural theory of the SEM approach. Lower panel shows the linear-regression approach. *Note*: The upper panel shows a schematic representation of the structural model testing the relationship between planning, patience, and risk preference and prospective reciprocity. Individual tasks are depicted in rectangular boxes. Latent constructs are depicted in circles. Unidirectional paths from latent construct to individual tasks represent factor loadings. Short blue arrows with pointed lines represent error variance. Unidirectional paths between latent constructs represent the relationships that will be tested. Dashed path and short arrow represent fixed parameters (factor loading and error variance) for the planning single item (Plan_Tower). The direction of arrows represents the causal relation from one construct to another construct or to the measurement instrument. The lower panel represents the situation where the individual prosocial tasks are predicted by the individual predictor tasks of risk, planning, and patience domains.

This multiple-task approach allowed us to investigate whether the data were consistent with a single underlying psychological construct related to forward-looking, sequential cooperation (as assumed in a structural equation modeling approach) while also allowing the possibility that the different tasks capture distinct facets of this construct that are better treated as separate predictors (as in a linear regression approach; see Fig. 1 for a schematic of both approaches). We deliberately included both description-based and experience-based tasks to mirror the variety of real-life decision contexts people face, which may differ in their cognitive demands.

## Methods

We pre-registered the methods of this paper prior to starting data collection. The preregistration can be viewed here: https://osf.io/prm5g.

### Participants

An a priori power analysis was performed to determine the required sample size for a Structural Equation Modelling approach. The power analysis was based on Root Mean Square Error (RMSEA) model-fit index, using the R package semPower^38,39^. The estimated required sample size (with 31 d.f., RMSEA = 0.06, α = 0.05 and β = 0.10) was *n* = 277.

Our final dataset comprised data from *n* = 297 participants. More information about inclusion criteria and how the final sample size was obtained is provided in the task descriptions and CONSORT diagram (Figure S9). Participants were recruited via the online platform Prolific Academic (https://www.prolific.com). We filtered for UK-based participants whose first language was English and who had not participated in any of our pilot experiments. As per the pre-registration, we excluded participants who failed to complete the entire battery, either because they chose not to continue or because they failed the inclusion criteria (as soon as a participant did not meet their first inclusion criterion, their entire data was dropped from the dataset and a new participant was recruited). We did not collect any personal data, and thus we do not report nor analyze further sample characteristics.

### Ethics information

Participants received compensation of £7.50/hour and could additionally earn task-specific bonus payments (see Table 1). Participants provided informed consent prior to participation. The study was approved by the University of Warwick Humanities and Social Science Research Ethics Committee (reference: HSSREC 165/19–20) and was conducted in accordance with relevant guidelines and regulations.

*Information regarding deception*. Participants were told in the general instructions (prior to providing their consent to participate) that some tasks would involve real interactions and some tasks would be hypothetical and that they would be debriefed, after the experiment, which tasks were the ones with real interactions. Of the interactive tasks, only the Dictator game involved a real interaction; specifically, the participants’ payoff was the amount that another real participant had sent in this task. In case this task was chosen by the bonus lottery at the end of the test battery, the participant received a bonus that was drawn from the pool of real Dictator game responses by the other participants. The “other” players in the other three interactive tasks (Trust game, Centipede game and Zürich Prosocial Game) were not real. The decisions of the other player in those tasks were pre-programmed. Research has shown that participants behave as if all tasks were real following such instructions and that behavior in real and hypothetical tasks is similar^40,41^.

### Design and procedure

All participants received the same order of tasks. We explicitly decided against counterbalancing the task order because we aimed at correlating individual differences in response profiles, making it important to provide all participants with the same stimuli, information, and order of tasks to minimize between-participant measurement noise^42^. This use of a fixed task order is a standard practice in studies of individual differences because counterbalancing would introduce an additional (unwanted) source of variation in the individual measurements^43,44^.

A session consisted of three or four tasks. Participants received their participation fee automatically after each session, once they submitted the respective link to Prolific. Table 1 provides a breakdown of how all tasks were incentivized, except the Kirby questionnaire. The Kirby questionnaire assesses whether participants prefer a smaller reward now or a larger reward in the future, but because Prolific does not allow payments to be that long delayed in time, this task was the only one that was not incentivized. Participants who completed the full test battery received an additional bonus payment based on the rewards obtained in three of the incentivized tasks. The bonus was explained at the beginning of each task, and participants learned how much bonus they earned at the end of each task. They also knew that the final bonus would be based on a lottery among all of their bonuses; particularly, the final bonus would be the sum of three bonuses, one chosen randomly from each study session. This incentive scheme involving a random selection of rewards gained in some of the tasks is a standard procedure in the behavioural sciences^32^. Table 1 presents an overview of the ten tasks that constituted the test battery. In the following, we provide a brief description of each task; for more details, please see supplementary materials.

**Table 1:** Task overview and composition of test sessions. Bonus Range indicates the observed range [Min-Max] of rewards that participants obtained in the different tasks.

Session	Task	(Short name)	Domain	Bonus Range (£)
1	Trust Game & Dictator Game (composite score)	(Coop_Trust)	Prosp. Reciprocity	Dictator [0–5]; Trust [5-7.50]
	Experience-based risky choice task	(Risk_Exp)	Risk	[3.96–5.27]
	Kirby monetary choice questions	(Pat_Kirby)	Patience	---
	Tower of London	(Plan_Tower)	Planning	[2.46–4.62]
2	Centipede Game	(Coop_Cent)	Prosp. Reciprocity	[0.5-5]
	Balloon Analogue Risk Task	(Risk_Bart)	Risk	[1.97–7.75]
	Hybrid Delay Task	(Pat_Hybrid)	Patience	[0.1–3.2]
3	Zürich Prosocial Game	(Coop_Zurich)	Prosp. Reciprocity	[0–4]
	Description-based risky choice task	(Risk_Desc)	Risk	[0.10–3.85]
	Boat task	(Pat_Boat)	Patience	[2.7–4.5]

### Tower of London (Plan_Tower)

The Tower of London task is a classic task to study planning ability^45,46^. Participants transform a given start state of three colored balls into a desired goal state in the minimum number of moves. We used the Tower of London “Freiburg” version, which is a systematically validated standard problem set that includes items with linearly increasing difficulty, based on a theoretical analysis of the problem space of the tower tasks^47–50^. Our outcome measure was the mean number of steps needed to solve the configurations of levels ‘5-steps’ and ‘6-steps’. We considered participants as dropouts if they produced timeouts in all 24 configurations.

### Kirby monetary choice questions (Pat_Kirby)

The Kirby questionnaire is an established measure of delay discounting^51–53^. Across 27 questions, participants were asked to indicate which reward they would prefer: the smaller reward today or the larger reward in the specified number of days. The outcome measure was the proportion of delayed choices. This task was not incentivized with a bonus. Three catch trials were interspersed where the smaller amount was the later amount. If participants choose this option more than once, they were considered dropouts.

### Hybrid delay task (Pat_Hybrid)

This task was modelled after an experience-based patience task for non-human primates^54–56^, which was developed to overcome potential confounds of two components of patience: inhibitory control during the choice between delays and the voluntary delay of gratification during delay maintenance. We developed this task in a previous study with humans^57^, and found that participants exhibited a consistent pattern of delay choice and delay maintenance. Because the task is less abstract and descriptive than the widely-used, questionnaire-based intertemporal choice tasks^52^, it makes a good candidate to provide a strong measure of human intertemporal preference. Participants played two rounds. In each round, they decided between a small-immediate reward option and a large-delayed reward option. If they chose the small option the reward was immediately transferred into their virtual bank. If they choose the large option an accumulation process started, during which the coins were transferred one by one into the bank until all coins were transferred or until the participant stopped the process. Each transfer took sixty seconds, which translated into a maximum duration of 16 min per round. The outcome measure was the number of collected coins.

### Boat task (Pat_Boat)

This is a simple experience-based patience task that we developed, inspired by Mies and colleagues^58^. In each round, participants decided which of two islands a boat should travel towards. The rounds differed in how many rewards could be collected on the islands and how far the boat had to travel to reach them. The farthest island was always the one with the highest payoff, whereas the closest island always had the lowest payoff. Participants played twelve rounds, which translated into a maximum duration of 20 min in case the larger reward was chosen in each round. In an additional catch round, the large reward was on the closer island and, to pass the catch round, participants had to pick the close island. The dependent measure was the proportion of regular rounds in which participants chose to travel the furthest (i.e., waiting the longest) to obtain the largest reward.

### Balloon analogue risk task (Risk_Bart)

The Balloon Analogue Risk Task (BART) is a computerized behavioral measure for risk taking^59^. In a recent large-scale evaluation of different risk measures, the BART was the behavioral measure that correlated most with propensity and frequency risk measures that are usually assessed via verbal reports^32^. In this task, participants pump up a series of balloons by clicking on a button on the screen. They have to decide if they want to pump up the balloon further or collect a reward based on the size of the balloon. The bigger the balloon, the bigger the reward. If the balloon pops, participants get nothing. Participants played twenty rounds. The outcome measure was the average adjusted number of pumps (defined as the average number of pumps excluding balloons that exploded). Participants were excluded if all balloons popped.

### Description-based risky choice task (Risk_Desc)

In this task, participants chose between pairs of described lotteries with varying degrees of risk^60^. Each pair consisted of a riskier (high variance) and a safer (low variance) option. With each successive pair, the expected value favored the riskier option more and more^60^. The dependent measure was the proportion of risky choices across all ten trials. Participants were excluded if they still picked the risky option in the last trial, which was a strictly dominated choice (i.e., both possibilities for the risky option were worse than the safe one).

### Experienced-based risky-choice task (Risk_Exp)

In this task, participants acquired information about the riskiness of choice options by experiencing the outcomes. This approach differs from ‘risk-from-description’ tasks, in which participants are explicitly told about the reward probability of an option (as above). The information format (experienced vs. described) has been shown to matter for people’s risk preferences in a number of studies^61–64^. Participants played three rounds of this task. In each round, they sampled from two options presented on screen and learned about the outcomes that were associated with each of them. Outcomes were determined by random draws from the pre-defined distribution underlying each option. Each round consisted of a riskier and a safer option and was presented fifty times in a row. The dependent measure was the proportion of risky choices in two rounds. The third round was a catch round, where the safe option always yielded zero. Participants were excluded if they picked this option more than 60% of the time.

### Zürich prosocial game (Coop_Zurich)

The task is a modified version of the Zürich Prosocial Game^37^. We developed new trial types that tapped into prospective reciprocity rather than retrospective reciprocity as in the original game. Participants received two trial types, prospective reciprocity and baseline, in a blocked design (six trials of each type). On prospective reciprocity trials, the other player needed help, and was potentially able to reciprocate helping later in the game. On baseline control trials, it was obvious that the other player would not be able to reciprocate later in the game. In each trial type, helping was costly for participants because they risked losing an item that they may potentially need for themselves later in the game. The outcome measure was the difference between helping in reciprocity trials minus helping in baseline trials. This score reflects the participants’ propensity to help strategically rather than altruistically (when the partner cannot reciprocate). Participants needed to answer at least two out of four comprehension questions correctly to be included and must have played (no time-outs) at least three trials per condition.

### Centipede game (Coop_Cent)

The Centipede game is played with two players who take alternating turns in deciding if they want to continue (cooperate) or stop (end the game)^34,36^. Prior to the first decision, both players received complete information about the payoffs at each decision point, including the final payoff they could receive if they make it through the whole game. By mutual cooperation until the final stage of the game, players could each reach a payoff that exceeded their individual payoffs at the start of the game. On any given decision point, however, if one player stopped the game, the other received less than they would have received at the previous point. We presented a test game with 20 decision nodes, in which there was substantial inequity between the two players’ payoffs at each node, and where reaching the final end point was less profitable than other previous nodes because there is a drop in payoffs at the very end (even though they are still higher than at the start). The measure of interest was the number of cooperative decisions, as a way of tapping into participants’ willingness to invest in a mutually beneficial interaction that can lead to higher payoffs for all but where there is also the risk of defection by the partner. The exclusion criterion in this task was a time-out if participants did not make any choice at all.

### Trust and dictator game (Coop_Trust)

Our participants played both a Trust game and a Dictator game. The **Trust game** is an established and extensively used experimental measure of trust in economics and psychology involving two players^19,65^. An “investor” is given a monetary endowment and can choose to send some of it to another player, the “trustee”. The amount sent is multiplied before it reaches the trustee. The trustee can send any proportion of the money back to return the favor, but there is no guarantee that they will. The **Dictator game** is similar to the trust game but without the possibility for the second player to send money back. The outcome measure for this task was the difference score between donations in the two games (donation amount in Trust game minus donation in Dictator game), which reflects how much of the investor’s behavior in the Trust game can be attributed to their strategic investment in the partner, i.e. expecting or hoping a return, rather than their prosocial preferences^66^. Participants had to respond correctly to at least two out of three comprehension questions that were asked prior to playing the Trust game.

### Analysis

We detailed the initial analysis plan in the pre-registration, where we planned to use Structural Equation Modelling (Plan A), or multivariate multiple regression (Plan B) in case the obtained data was unsuitable for the former. The pattern of correlations observed in the data among the three measures of strategic future-oriented reciprocal cooperation (Coop_Trust, Coop_Cent, Coop_Zurich) and the different indicators of the three cognitive variables (Plan_Tower, Pat_Hybrid, Pat_Kirby, Pat_Boat, Risk_Bart, Risk_Exp, Risk_Desc) ranged merely from low to moderate (see Table 2). This finding led us to discard the initially proposed SEM approach.

Instead, three multiple regressions were conducted, one for each of the measures of prospective reciprocity as the dependent variable. In each model, we used bootstrap (*N* = 1000) to predict the respective dependent variable from the seven predictor tasks: Plan_Tower, Pat_Hybrid, Pat_Kirby, Pat_Boat, Risk_Bart, Risk_Exp, and Risk_Desc. For the Centipede game, we additionally employed a zero-one-inflated beta regression model (ZOIBR)^67^ due to the obtained distribution of responses (many people defected either at the first decision, or cooperated until the end). Both regression analyses revealed congruent results in the sense that the same predictors account for variation in the dependent variable, but the ZOIBR model specified which part of the data (0/1 responses or the continuous part) was associated with the predictors. We present the ZOIBR results here, because this model fits the data better and gives more precise information about the role of the predictors. We additionally report results of the linear regression in the supplementary information, as this was the pre-registered approach. The ZOIBR was implemented within a Bayesian framework using the brms R package^68^.

In addition to unstandardized and standardized coefficients, structure coefficients, Johnson’s relative importance weights (RIW)^69,70^ and the relative importance (in %) of the predictors based on RIW were calculated (see ESM for more details).

Furthermore, in correlation and regression analyses, unreliable measurements can result in underestimated relationships and elevate the risk of Type II errors. This is especially relevant when the research objective involves capturing the inherent relationships present within the population with precision. While reliability estimates (such as Cronbach’s alphas) ranging from 0.7 to 0.8 are acceptable^71^, measurements of even this reliability encompass enough measurement error to necessitate correction. We thus replicated the multiple regression analyses incorporating a correction for attenuation to achieve a more accurate representation of the true relationships between variables.

We provide a more detailed description of the performed analysis, coefficients, task reliabilities, and corrections for attenuation in the supplementary information.

## Results

**Fig. 2:**
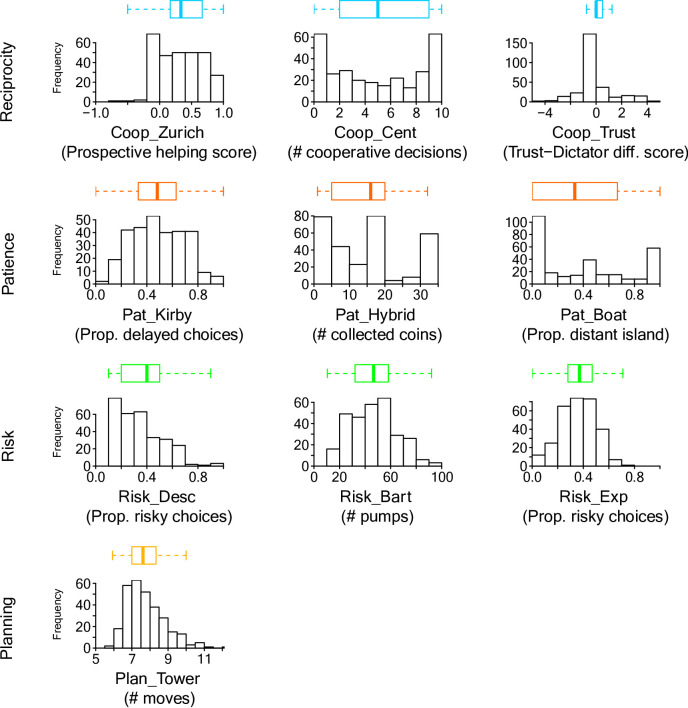
Overview of participant responses. Each plot shows the frequency of responses across the response space of the respective task. Along the top is a sideways box plot displaying the median and quartiles; whiskers extend to the smallest and largest values within 1.5 times the interquartile range of the respective quartiles, and points beyond this range are considered outliers.

Most tasks yielded a good spread of responses along the respective dimensions of interest, making the data suitable for detecting individual differences (see Fig. 2).

### Correlations

Table 2 presents the observed Pearson correlation coefficients between outcome variables and predictors.

**Table 2:**
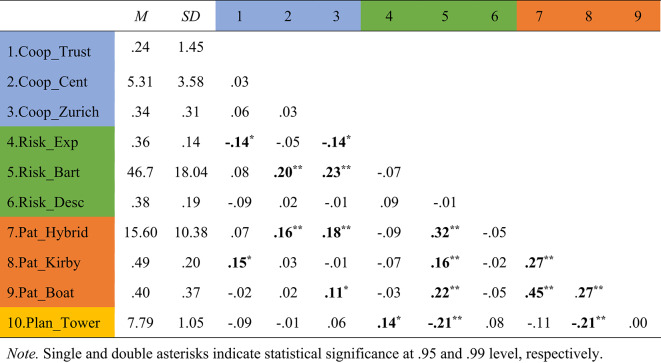
Means, Standard deviations, and Pearson correlation coefficients.

Note. Single and double asterisks indicate statistical significance at 0.95 and 0.99 level, respectively.

### Zürich Prosocial Game

**Fig. 3:**
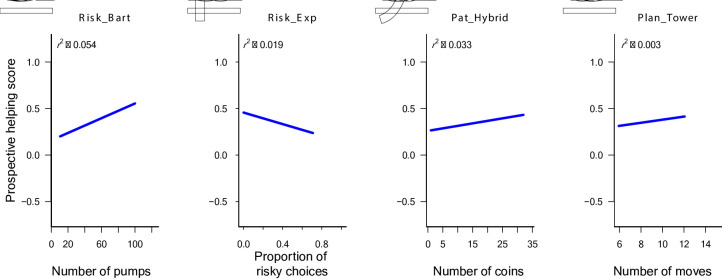
Scatterplots and regression lines for the four best predictor variables of the Zürich Prosocial Game (Coop_Zurich). Depicted are bivariate scatter plots with respective least-squares regression lines and r^2^.

The distribution of responses indicates that participants tended to help more in prospective reciprocity trials compared to baseline trials (see the mostly positive Coop_Zurich scores in Fig. 2), suggesting a strategic component in their helping behaviour. Four predictors contributed significantly to explaining the variance in the model (see Table 2 and Table 3). Risk_Bart showed a statistically significant positive standardized coefficient (*Beta* = 0.21, *p* < .001). Hence, riskier behavior (higher Risk_Bart scores) was associated with more strategic helping (higher Coop_Zurich scores). In contrast, riskier behavior in Risk_Exp (higher scores) was associated with less strategic helping (*Beta* = − 0.13, *p* = .011). Furthermore, better planning performance (lower Plan_Tower scores) was moderately associated with less strategic helping (*Beta* = 0.12, *p* = .049). Finally, more patience (higher Pat_Hybrid scores) was moderately associated with more strategic helping (*Beta* = 0.12, *p* = .055), though marginally not significant at the conventional 0.05 threshold (but see results for disattenuated models in ESM). Figure 3 visualizes the relationships of these four predictors with the Zürich prosocial game. Overall, the model accounted for 10.1% of the variance in Coop_Zurich score (${R}^{2}$= 0.101), with an *adjusted* ${R}^{2}$ of 0.08 (${F}_{7,289}$ = 4.636, *p* < .001).

**Table 3:** Multiple regression coefficients for predicting Coop_Zurich score.

	Unstandardized Coefficients		Standardized Coefficients	Bootstrap Std. Error	Bootstrap Sig, (2-tailed)	Bootstrap 95% Confidence Interval for B	
	B	Std. Error	Beta			Lower Bound	Upper Bound
(Constant)	0.000	0.165		0.175	0.999	− 0.369	0.331
**Risk_Exp**	**− 0.299**	**0.127**	**− 0.133**	**0.116**	**0.011**	**− 0.529**	**− 0.080**
**Risk_Bart**	**0.004**	**0.001**	**0.214**	**0.001**	**< 0.001**	**0.002**	**0.006**
Risk_Desc	0.008	0.090	0.005	0.090	0.936	− 0.165	0.184
*Pat_Hybrid*	*0.004*	*0.002*	*0.122*	*0.002*	*0.055*	*0.000*	*0.007*
Pat_Kirby	− 0.110	0.093	− 0.071	0.093	0.233	− 0.291	0.066
Pat_Boat	0.023	0.053	0.027	0.053	0.659	− 0.079	0.121
**Plan_Tower**	**0.034**	**0.017**	**0.118**	**0.018**	**0.049**	**0.001**	**0.071**

Note. Bold text indicates statistically significant coefficients (*p* < .05), and italics indicates a trend (*p* < .1).

### Centipede Game

**Fig. 4:**
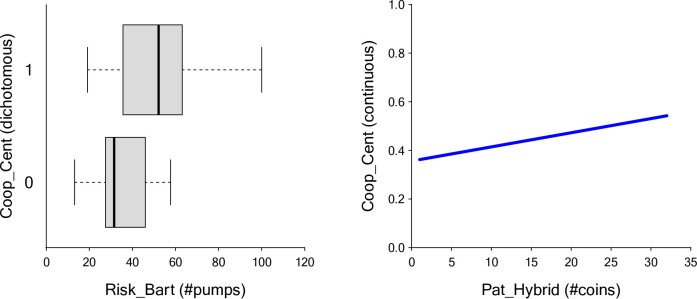
Results of the ZOIB regression for the Centipede game. *Note*: The boxplot on the left shows the discrete part of the model for Risk_Bart scores among participants who never cooperated (0, *n* = 22) and those who always cooperated (1, *n* = 63). The plot on the right depicts the continuous part of the model for Pat_Hybrid scores among participants who had cooperated at different levels (*n* = 212).

The Centipede game yielded a distribution where a high number of people either never cooperated or always cooperated (see Fig. 2) and was thus analyzed with a zero-one-inflated beta regression (ZOIBR). The analysis revealed that the Risk_Bart task was linked to the dichotomous aspect of the model, distinguishing participants who never cooperate from participants who always cooperate (estimate = 0.08, SEM = 0.02, CI(95%)[0.04,0.12]). Higher Risk_Bart scores increased the likelihood of full cooperation (Fig. 4). Within the same model, Risk_Bart scores were not related to the continuous part of the model for individuals who cooperate at varying levels (estimate = 0.01, SEM = 0.01, CI(95%)[-0.01,0.02]). In contrast, the Hybrid-delay Task (Pat_Hybrid) was associated with the continuous part of the model, specifically for individuals who cooperated at different levels (estimate = 0.02, SEM = 0.01, CI(95%)[0.00,0.04]. Among those participants who engaged in cooperation to some degree, those with higher Pat_Hybrid scores were more inclined to cooperate at higher levels. The Bayesian pseudo-${R}^{2}$ value for the model was 0.075, indicating the proportion of variance explained by the model. Table S12 displays the recovered posterior means, standard deviations, and 95% credible intervals.

### Trust

**Fig. 5:**
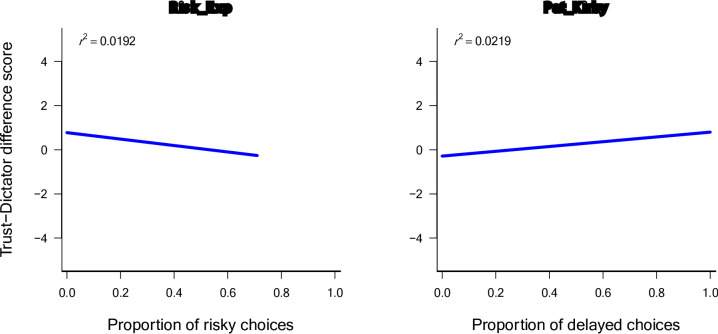
Scatterplots and regression lines for the two best predictor variables of the Trust-Dictator difference score (Coop_Trust).

The distribution of Trust-Dictator difference scores indicates that most participants gave the same amount in the Trust game and the Dictator game (see the Coop_Trust scores clustering around zero in Fig. 2). Two of the predictor measures demonstrated statistically significant relations with the Coop_Trust score (see Table 4). More patience (higher Pat_Kirby scores) was associated with more strategic investments (higher Coop_Trust scores, *Beta* = 0.138, *p* = .031). More risky decisions (higher Risk_Exp scores) were associated with less strategic investment (*Beta* = − 0.114, *p* = .040). Figure 5 visualizes the relationships of the two best predictors with the Trust-Dictator game difference score. The overall model was statistically significant (${F}_{7,289}$ = 2.293, *p* = .027) and accounts for 5.3% of the variance (${R}^{2}$ = 0.053). However, the *adjusted* ${R}^{2}$ value was only 0.03.

**Table 4:** Multiple regression coefficients for predicting Coop_Trust scores.

	Unstandardized Coefficients		Standardized Coefficients	Bootstrap Std. Error	Bootstrap Sig. (2-tailed)	Bootstrap 95% Confidence Interval for B	
	B	Std. Error	Beta			Lower Bound	Upper Bound
(Constant)	0.588	0.799		0.724	0.420	− 0.777	2.065
**Risk_Exp**	**-1.198**	**0.614**	**− 0.114**	**0.567**	**0.040**	**-2.292**	**− 0.075**
Risk_Bart	0.004	0.005	0.046	0.005	0.451	− 0.006	0.015
Risk_Desc	− 0.580	0.435	− 0.077	0.422	0.161	-1.418	0.174
**Pat_Kirby**	**1.003**	**0.449**	**0.138**	**0.457**	**0.031**	**0.159**	**1.953**
Pat_Boat	− 0.325	0.225	− 0.084	0.269	0.229	− 0.835	0.205
Pat_Hybrid	0.005	0.009	0.034	0.011	0.683	− 0.017	0.026
Plan_Tower	− 0.038	0.083	− 0.028	0.085	0.664	− 0.217	0.117

Note. Bold text indicates statistically significant coefficients (*p* < .05).

**Table 5:** Overview of observed association patterns.

Task	Effect on Coop_Zurich	Effect on Coop_Cent	Effect on Coop_Trust
**Risk_Exp**	More strategic help is associated with ***less*** risky choice	--	More strategic investment is associated with ***less*** risky choice
**Risk_Desc**	--	--	--
**Risk_Bart**	More strategic help is associated with ***more*** risky choice	More cooperation is associated with ***more*** risky choice	--
**Pat_Kirby**	--	--	More strategic investment is associated with ***more*** patience
**Pat_Boat**	--	--	--
**Pat_Hybrid**	More strategic help is associated with ***more*** patience (trend)	More cooperation is associated with ***more*** patience	--
**Plan_Tower**	More strategic help is associated with ***less*** planning ability	--	--

## Discussion

We found that several psychological traits predicted participants’ behavior in three prospective reciprocity tasks (see Table 5). Specifically, patience was positively related to strategic investments in all three reciprocity tasks. This finding is in line with our theoretical prediction that people who exhibit more patience may show more prospective reciprocity than less patient people. It is also consistent with recent findings in preschoolers, where greater willingness to delay rewards was associated with the tendency to invest in potential reciprocators^72^. Such strategic investment was only apparent in 5-year-olds, but not younger children. Yet, even 5-year-olds developed strategic sharing patterns only after several trials, indicating that the children seemingly learned about the benefits of investing in a co-player but did not yet engage in prospective reciprocity spontaneously. Prospective reciprocity thus appears to develop relatively late in children, probably due to its cognitive demands^15^. The findings are also in line with previous studies showing a positive correlation between patience and other forms of cooperation in adult humans. For example, Al-Ubaydli and colleagues^73^ found that pairs of individuals who were on average more patient were more likely to coordinate on the socially efficient outcome in a stag-hunt game, obtaining higher payoffs. Harris and Madden^25^ found that patience was positively correlated with cooperation in a prisoner et al.^75^). In these previous games, cooperation decisions were made simultaneously, and our results extend these prior findings to situations in which individuals must decide whether to invest in a partner, who can in principle reciprocate in the near future. An interesting direction for future research would be to investigate whether measures of patience are similarly predictive of other forms of forward-looking cooperation, such as indirect reciprocity or prosocial acts aimed at enhancing one’s reputation.

We did not find the expected positive relation between planning and prospective reciprocity. Performance in the planning task was only significantly related to one reciprocity measure, namely the Zürich Prosocial Game. Participants who performed better in the planning task engaged in less strategic helping, which is the opposite pattern than we expected. Rand and colleagues^75,76^ suggested that reflection may moderate cooperative behaviours and demonstrated in a series of experiments that faster decisions were consistently associated with more cooperation. In addition, manipulating time pressure and conceptual priming of intuition vs. reflection led to the same pattern of variation in cooperative decisions. People cooperated more when they were making more intuitive decisions and less when reflecting more. Subsequent replication studies of the effect of time pressure on cooperation failed^77^, however, and a study with ultimatum game responses and cognitive load found that reduced cognitive capacities merely increased decision noise, but did not systematically affect social preferences^78^.

Grueneisen and colleagues^12^ recently found that children who showed better prospection skills were more likely to invest in partners who could later reciprocate sharing toys than children with weaker prospection skills. One possibility to reconcile these findings with the current findings is that the relation between planning abilities and future-oriented reciprocity may not follow a continuous pattern but rather reflect a step function: A minimum amount of planning and thinking ahead is necessary to engage in future-oriented reciprocity, but beyond this threshold, better planning abilities do not lead to more reciprocity. One could argue that the planning required for prospective reciprocity is just about thinking one step ahead and anticipating the help needed from the partner, so that minimal future or long-term thinking suffices^10^.

Another explanation for our results is that better planning skills might lead people to focus on the possibility of being exploited, as classical rational theory would predict. For example, if participants in the ZPG accurately predicted that there were not any further interactions with the same partner and reasoned that this was also common knowledge to their partners, they may have expected partners to behave selfishly and not reciprocate. This anticipated exploitation would explain the lower levels of strategic helping observed in participants who were better at planning.

Finally, better planning skills may only be relevant for reciprocity when the time delay between favors exchanged is not a few minutes, as in our study, but rather days, or weeks. Future studies could try to obtain additional measures about the level of future thinking and strategizing by participants in the reciprocity tasks, as well as present participants with various tasks that operationalize “planning” in different ways.

The pattern for the risk domain was less clear. One risk index (Risk_Bart) was positively related with two of the prospective reciprocity measures (Centipede game and Zürich prosocial game), a pattern that is in accordance with our hypothesis that people who can tolerate or seek more risk will show more future-oriented cooperation. In contrast, another risk index (Risk_Exp) was negatively related with two prospective reciprocity measures (Trust-Dictator difference score and Zürich prosocial game), thus showing the opposite pattern. This divergence is particularly interesting in light of previous findings where different measures of risk yielded different responses within the same participants^32^, a pattern that was partly attributed to differences in the tasks’ choice architecture, i.e., how risks, rewards, and the consequences of an error – choosing the options with the lower expected utility, interact with one another^79^. Hence, a possible explanation for these diverging association patterns could be that different motives are at play in the different risk tasks: particularly, high scores in Risk_Bart might not purely reflect participants’ high risk tolerance but might also be related to reward-maximizing motives, because there is a direct relation between the number of pumps and the size of the reward^79^. In the Risk_Exp task, in contrast, both options have the same overall expected value while still differing in risk (probability to win/lose). Hence, the positive relation between Risk_Bart and Coop_Zurich may be (partly) driven by payoff maximization, whereas we can exclude this confounding factor to explain the negative relation between Risk_Exp and Coop_Zurich. Consequently, one may argue that the latter reflects a “purer” relationship between risk tolerance and prospective reciprocity. In this case, the resulting relationship is the opposite of what we predicted, meaning that lower tolerance to risk or more risk aversion is related to more strategic helping in the Zurich Prosocial game.

The individual differences found in time and risk preferences could be based on contextually-appropriate responses to one’s ecology as argued by Boon-Falleur et al. ^81^ The argument is that access to varying amounts of material resources may have behavioural and psychological consequences. Having more resources and being able to satisfy pressing needs allows individuals to become more future-oriented and maybe more risk tolerant. In reciprocal cooperation when benefits accrue only over time, individuals with more pressing needs may be more cautious and less able to wait and risk not receiving reciprocal benefits down the line^24,80^. There is empirical evidence showing that access to resources is associated with greater cooperation^81,82^. While this aspect cannot be analyzed further because no information regarding participants’ ecological context or access to material resources was collected, this possibility represents an interesting avenue for future research.

Surprisingly, most participants gave the same amounts in the Trust game and Dictator game, as is apparent from the Coop_Trust score clustering around zero with very little variation (see Fig. 2). Previous research has consistently found differences between the two games with more sharing in the Trust game, suggesting that the extra sharing, in comparison to the dictator game, is motivated by the expectation of a return^83,84^. Most previous research comparing the two games has used between-subjects designs, however. Ashraf and colleagues^66^ conducted a within-subjects study like ours and found that the order of the two conditions had an effect on participants’ responses, and that conducting the Trust game first led to higher investments in the Trust game and a difference with the Dictator game. This study is the reason why we also chose that same order (Trust game first), but we cannot exclude the possibility that order effects in our within-subjects design may account for the lack of variation between Trust and Dictator games. For now, we can only conclude that, within the limits of our test battery, the composite Trust score did not yield as much variation as expected. To reduce the risk of carry-over effects, future studies may want to consider presenting the two games in separate test sessions.

The test battery included both description-based and experience-based tasks. We deliberately included behavioral measures as they are closer to people’s real-life experiences^64^ than questionnaires and hypothetical tasks, and can be used in a broader variety of study populations, for example with young children and non-human animals. This variety of tasks may have contributed to some of the weak and unexpected correlations among some of the tasks. Correlations among tasks of the same domain were only significant for patience. The absence of correlations within the other domains indicates that different measures may tap into different components of the respective constructs and/or that different tasks elicit different responses due to the contexts in which the decisions are made^79^.

Importantly, the absence of correlations among the three reciprocity tasks suggests that prospective reciprocity may not be well captured as a single underlying construct. Rather, the tasks may be better understood as a group of cooperative decisions that share a common functional structure: namely, investments in others made under conditions that allow for anticipation of future returns. From this perspective, the lack of shared variance across tasks highlights the context sensitivity of forward-looking cooperation. Specifically, such cooperation appears to rely on partially distinct cognitive and motivational processes, depending on task structure and situational demands. From a psychometric perspective, our findings suggest that prospective reciprocity may be conceptualized as a formative rather than reflective construct. In a reflective measurement model, which assumes that task scores are alternative manifestations of a single underlying latent variable, indicators should correlate because they are caused by the same source. In contrast, formative models do not require indicator correlations because the indicators represent different causal inputs that combine to constitute the construct. Under this interpretation, planning, patience, and risk tolerance may combine in task-specific configurations to support prospective reciprocity, with different tasks weighting these components differently depending on their specific demands.

Similar patterns of weak cross-task correlations have been documented within the cooperation and social preferences literature, where different behavioural measures of theoretically coherent constructs, such as fairness, generosity or reciprocity, often show limited convergence^85,86^. For example, Yamagishi and colleagues^85^ report weak convergence among measures commonly interpreted as reflecting a preference for fairness or reciprocity. Specifically, they find no correlation between rejection of unfair offers in the Ultimatum Game and positively reciprocal behaviour in other tasks, such as the Trust Game or the Dictator Game.

The possibility that prospective reciprocal behaviour is highly context-dependent was one of the reasons we adopted a multiple-task approach. For example, the Zürich Prosocial Game is much more dynamic than the Trust game, where participants merely contemplate how much money to invest in a potential reciprocator, who can reciprocate a few seconds later. In addition, in the Trust game participants make decisions about sharing money, whereas in the ZPG participants help by donating a tool (key) which can affect their own possibilities of winning the game. On a conceptual level, both tasks are meant to assess strategic investments in others. The tasks, however, differ greatly in what participants have to pay attention to while playing them, with the Zürich Prosocial Game having a lot of additional information (e.g., remaining time, key endowments of both players, remaining number of doors of different colors, probabilities of needing future help and being able to help) that is not directly related to the helping decision. Thus, as a field we might have to acknowledge that either behavioural measures of forward-looking reciprocity depend a lot on task specificities, or we need to define the construct much more narrowly so that it becomes synonymous with a certain behavioral task. Here, we followed the first approach and think future work should further explore different tasks of forward-looking reciprocity and try to explain behaviors in them.

The task battery used a combination of new and old tasks, which worked to varying degrees. In general, the Zürich Prosocial Game and the Centipede game, i.e., the two measures which have not been as widely used as the Trust game, worked well. There were correlations between these two cooperation measures and several of our other measures, in particular patience (Pat_Hybrid) and risk (Risk_Bart). For both tasks, we found correlations with all three psychological mechanisms. The fact that the Zürich Prosocial Game was related to four tasks spanning all domains makes it a promising reciprocal cooperation task for further studies. The Centipede Game represents a natural sequential form of interacting cooperatively with others and correlated with experience-based measures of risk and patience. Hence, the Centipede Game is also a promising and interesting measure to study reciprocal cooperation and its psychological underpinnings. The two predictor tasks that were not associated with any of the outcome measures were the well-established and widely used description-based risky choice task (Risk_Desc) on the one hand and a novel patience task (Pat_Boat) on the other hand. Among the novel or customized tasks, the Hybrid-Delay task (Pat_Hybrid) and the experience-based risky choice task (Risk_Exp) seemed to be a viable measure of individual differences, as evidenced by the results of correlations with different reciprocity measures.

### Conclusions and future directions

Across three prospective reciprocity tasks, performance showed little convergence, indicating that forward-looking cooperative behavior may not be well captured as a unitary, domain-general trait. The present results therefore caution against interpreting prospective reciprocity as a single latent disposition and instead highlight the importance of task structure and situational demands in shaping forward-looking cooperative behavior.

At the same time, measures of planning, patience, and risk tolerance were related to performance within these cooperative tasks in task-specific ways, with no single psychological mechanism accounting for behaviour across all contexts. The absence of consistent domain-general association patterns has important implications for the study of cooperation in behavioral economics and psychology. Our findings warrant caution when interpreting findings based on single behavioral elicitation methods and highlight the need for a better psychometric understanding of prospective reciprocity and other psychological constructs.

### Data Availability

Data is available here: https://osf.io/hnwjs/?view_only=97484df47e9f48b0972d0cf2b6ef73f3.

#### Code availability statement

Code for the tasks will be made available upon publication.

## Supplementary Information

Below is the link to the electronic supplementary material.

Supplementary Material 1

## Data Availability

Data is available here: [https://osf.io/hnwjs/?view_only=97484df47e9f48b0972d0cf2b6ef73f3](https:/osf.io/hnwjs/?view_only=97484df47e9f48b0972d0cf2b6ef73f3).

## **Terminology (Box 1)**

### Direct positive reciprocity

Mutually beneficial cooperative interaction between two individuals consisting of a contingency-based exchange of cooperative favours over time, such that A benefits B (at a possible personal cost) and B returns the favour later in time (at a possible personal cost).

### Retrospective reciprocity

Reciprocity based on individuals keeping track and responding to others’ previous behaviours, preferentially cooperating with indviduals who were helpful in the past. The exact proximate mechanism sustaining this form of reciprocity can vary and be based on emotional bookkeeping or “attitudinal reciprocity”, which are mechanisms whereby individuals act altruistically preferentially towards social partners with whom they have close social bonds and share a positive emotional history. Retrospective reciprocity, however, may also be based on a more cognitive mechanism (“calculated reciprocity”) where individuals remember the exact number of favours given and received with other social partners.

### Prospective reciprocity

Reciprocity based on individuals investing in others in anticipation of future payback, with cooperative acts in the present being motivated by the possibility of future reciprocation. The level of intentional strategizing in prospective reciprocity could in principle vary. In its simplest form, individuals could feel a stronger bond toward others with whom they presume to interact again in the future, eliciting prosocial acts in the absence of explicit expectations of others’ subsequent behaviors. At a minimum, however, prospective reciprocity requires the capacity to identify or anticipate situations that present opportunities for reciprocation. Alternatively, at the other extreme, prospective reciprocity could be based on individuals’ capacity to think ahead, and deliberately consider the social consequences of their actions, so that current cooperation is motivated by the expectation of returned benefits.
