# Supplementary material for: The role of future planning, patience, and risk tolerance for prospective reciprocity in human adults
Source: Sci Rep. 2026 Mar 6;16:12383. doi: 10.1038/s41598-026-42226-3 (PMC13083854; doi:10.1038/s41598-026-42226-3)
Supplement: Supplementary file 1 — Supplementary Material 1 [file 41598_2026_42226_MOESM1_ESM.docx]

*Supplementary Information* for

The role of future planning, patience, and risk tolerance for prospective reciprocity in human adults

Stefanie Keupp, Sebastian Grüneisen, Sebastian Olschewski, Maria Victoria Hernández-Lloreda, Felix Warneken, Elliot A. Ludvig, and Alicia P. Melis

[Tasks 2](#_Toc168581373)

[Planning 2](#_Toc168581374)

[Tower of London (Plan_Tower) 2](#_Toc168581375)

[Patience 3](#_Toc168581376)

[Kirby monetary choice questions (Pat_Kirby) 3](#_Toc168581377)

[Hybrid delay task (Pat_Hybrid) 4](#_Toc168581378)

[Boat task (Pat_Boat) 5](#_Toc168581379)

[Risk 7](#_Toc168581380)

[Balloon Analogue Risk Task (Risk_Bart) 7](#_Toc168581381)

[Experienced-based risky choice task (Risk_Exp) 8](#_Toc168581382)

[Description-based risky choice task (Risk_Desc) 9](#_Toc168581383)

[Reciprocity measures 10](#_Toc168581384)

[Zürich Prosocial Game (Coop_Zurich) 10](#_Toc168581385)

[Centipede game (Coop_Cent) 12](#_Toc168581386)

[Trust/Dictator game (Coop_Trust) 14](#_Toc168581387)

[Participants and Procedure 16](#_Toc168581388)

[Additional information on analysis and results 17](#_Toc168581389)

[General approach 17](#_Toc168581390)

[Coefficients 18](#_Toc168581391)

[Zero-inflated Beta regression (ZOIBR) 19](#_Toc168581392)

[Correction for attenuation 19](#_Toc168581393)

[Results 20](#_Toc168581394)

[Correlations and reliability coefficients 20](#_Toc168581395)

[Multiple regressions 21](#_Toc168581396)

[Zürich Prosocial Game 21](#_Toc168581397)

[Centipede Game 25](#_Toc168581398)

[Trust 28](#_Toc168581399)

[References 29](#_Toc168581400)

# Tasks

We ran short pilot studies to test the feasibility of the tasks and to determine appropriate specifications. The goal was to find task specifications that resulted in variation of participant responses as well as testing mechanics of the tasks, some of which have not been used in online formats before. If participants responded too uniformly (e.g., revealing clear ceiling or floor effects), we adapted the respective task in the needed direction (for example, increasing/decreasing delays, success probabilities, and bonus rewards). Participants were recruited from the same *Prolific* participant pool (www.prolific.co) and were paid according to the same reward scheme as was used for the main study. They were informed about the participation fee and the possibility of bonus payments, prior to data collection.

## Planning

### Tower of London (Plan_Tower)

The Tower of London task is a classic task to study planning and executive function abilities^1,2^. The task is to transform a given start state of three colored balls into a desired goal state with the minimum number of moves (see Figure S1). Certain rules apply: only one ball can be moved at a time, balls cannot be placed outside the rods, and length of the rods restricts how many balls can be stacked on it.

| **Figure S1**  *Screenshot of a Tower configuration requiring a minimum of four moves to reach the goal state.* |
| --- |
| 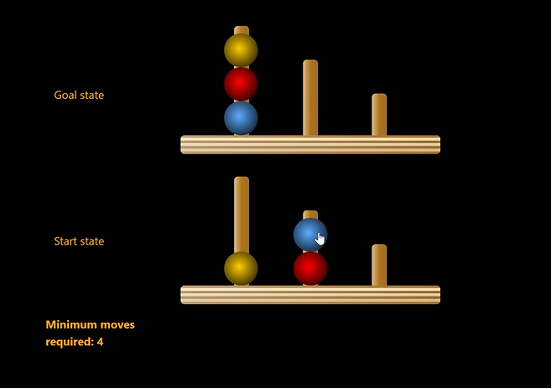 |

We used the combinations of the Tower of London from the “Freiburg version”. This is a systematically validated standard problem set that includes items with linearly increasing difficulty, based on theoretical analysis of the problem space of the tower tasks^3–6^. It is validated in different populations and can detect stable individual differences. This set of configurations is balanced regarding the following dimensions: (i) minimum number of moves required, (ii) search depth (i.e., number of intermediate moves that have to be considered before the first goal move can be reached), and (iii) goal hierarchy (i.e., ambiguity of the sequence of final moves derived from the configuration of the goal state (for more information, see also Table 1 in Kaller et al.^3^).

We presented the full set of 24 configurations^3^ to our participants but did not include the eight easy configurations (‘4-steps’) in our analysis. Our pilot sample (*n* = 16) showed ceiling effects with the easy configurations, but as the set is validated for the full 24 configurations, we decided not to deviate from the original methods by presenting only a subset of configurations. Participants had up to 60 seconds to solve each configuration. Our outcome measure was the mean number of steps needed to solve the configurations of categories ‘5-steps’ and ‘6-steps’.

**Dropout criterion:** Participants were excluded if they produced a time-out in all 24 configurations.

## Patience

### Kirby monetary choice questions (Pat_Kirby)

The Kirby questionnaire is an established measure of delay discounting^7–9^. Across 27 questions, participants were asked to indicate which reward they would prefer: the smaller reward today, or the larger reward in the specified number of days. For example: “Would you prefer £54 today or £55 in 117 days?”. Items were presented individually with the next item to appear after participants made their choice on the previous item. Choices were displayed as two buttons with specified reward and delay time on each button (see Figure S2). Participants indicated their choice by clicking on their preferred option.

| **Figure S2**  *Screenshot of a Kirby question.* |
| --- |
| 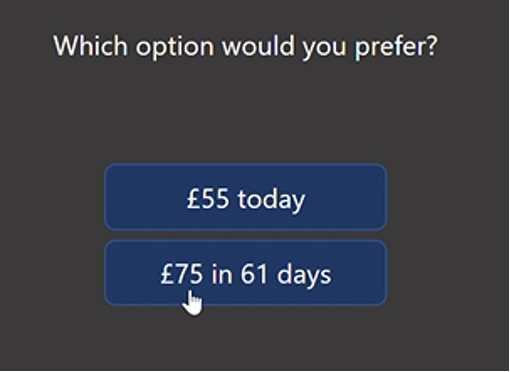 |

Participants were informed prior to answering the questions that this task was not incentivized. We chose to not incentivize this task because it was not possible to pay individual bonus rewards from this particular task at different time points, i.e., corresponding to the delay period specified in each item. While individualized payment schemes may be feasible when administering this task as a stand-alone experiment or when recruiting participants from a local subject pool, we found it difficult to realize such schemes as the task was part of an online test battery with several sessions and using a commercial recruitment platform (Prolific required to pay participants within 21 days). We interspersed three catch trials, where the amount one can get today is larger than the amount one can get in the future.

Different ways to calculate individual delay discounting scores are possible: (i) scoring procedure as described in Kirby et al.^9^ to estimate discounting rate (value of the k-parameter in a hyperbolic discounting function), or (ii) logistic regression to estimate discounting rate^10^, or (iii) proportion of choices of delayed rewards^11^. Myerson et al.^11^ compared the three possibilities and recommended proportion of delayed choices as an easy-to-calculate, valid, reliable measure that is free of theoretical assumptions. We followed this recommendation and used proportion of delayed choices as our outcome measure.

**Dropout criterion:** Participants were excluded if they answered more than one out of three catch questions incorrectly, i.e., indicating they wanted to wait longer for a smaller option.

### Hybrid delay task (Pat_Hybrid)

This task was modelled after an experience-based patience task for non-human primates^12–14^, which was developed to overcome potential confounds of two components of patience: inhibitory control during initial choice and voluntary delay of gratification during delay maintenance. In the non-human-primate version of the classic delay of gratification task, individuals get to choose between a smaller-sooner and a larger-later reward option. If they choose the smaller option, they receive it immediately. If they choose the larger option, they have to wait a predetermined period of time until they receive the reward; crucially, there is no chance to reverse an initial larger-later choice (for example, in case it was made by reflexively pointing to larger amounts of food). In contrast, the Hybrid-delay task lets individuals correct their choice: If the large option was chosen, an accumulation process starts during which food items are transferred one by one within reach of the individual. The individual can abort the process and consume the amount of food that has accumulated on their end up until this point. This task has not been used in humans before but given its power to dissociate a preference for large-delayed rewards from the ability to maintain delays, and given that it is less abstract and descriptive than the widely-used, questionnaire-based intertemporal choice tasks^8^, we reasoned it would make a good candidate to provide additional insights into human intertemporal choices.

Our version of the task for human adults took place online and used a virtual reward coin currency that translated into bonus rewards. Participants could choose between a small-immediate reward option (four coins) and a large-delayed reward option (16 coins). If they chose the small option, the reward was immediately transferred into their virtual bank and the next choice was presented. If they chose the large option, an accumulation process started during which the coins were transferred one by one into the bank (see Figure S3). Each transfer took 60 seconds. Participants played two rounds, hence they had to wait 32 minutes in order to obtain the full reward amount of 32 coins (16 minutes per round). At any point, however, they could stop the accumulation process and move on. In this case, they did not receive the full 16 coins but only the number they had accumulated up to that point. A secondary task ensured that participants remained focused on the experiment: a square appeared at random intervals in a specific position on the screen and participants had to click on it within 10 seconds. In case they missed this time window, they received a timeout message and did not receive any bonus for this round. Our measure of interest is the total number of collected coins per participant.

| **Figure S3**  *Screenshot of the Hybrid Delay Task.* |
| --- |
| 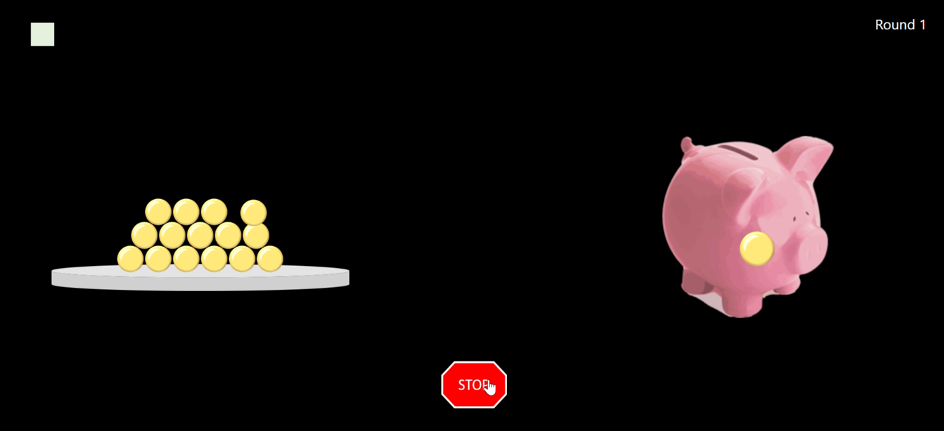 |

**Dropout criterion:** Participants were excluded if they failed the attention check (the secondary task) in both rounds.

### Boat task (Pat_Boat)

The “Boat task” is a simple experience-based patience task that we developed, inspired by Mies and colleagues^15^. On each round, participants decided which of two islands a boat should travel to (see Figure S4). The rounds differed in how many rewards could be collected on the islands and how far the boat had to travel to reach them. The further island was always the one with the highest payoff (6 or 8 rewards), whereas the closer island always had the lowest payoff (4 rewards). Participants played twelve rounds, in which the payoffs and distance to the two islands were varied. In an additional catch round, the large reward was on the closer island. The dependent measure was the proportion of rounds in which participants chose to travel further (i.e., waiting longer) to obtain the larger reward. Depending on starting point, the boat took 90 s, 100 s, or 110 s to reach the island with the larger payoff. The corresponding journeys to the island with the smaller payoff took 10 s, 20 s, or 30 s. As we wanted to avoid having to interpret change-of-direction events, participants were instructed that they must decide their goal before they make the first move. They could not change direction anymore after their first move.

| **Figure S4**  *Screenshot of the Boat task.* |
| --- |
| 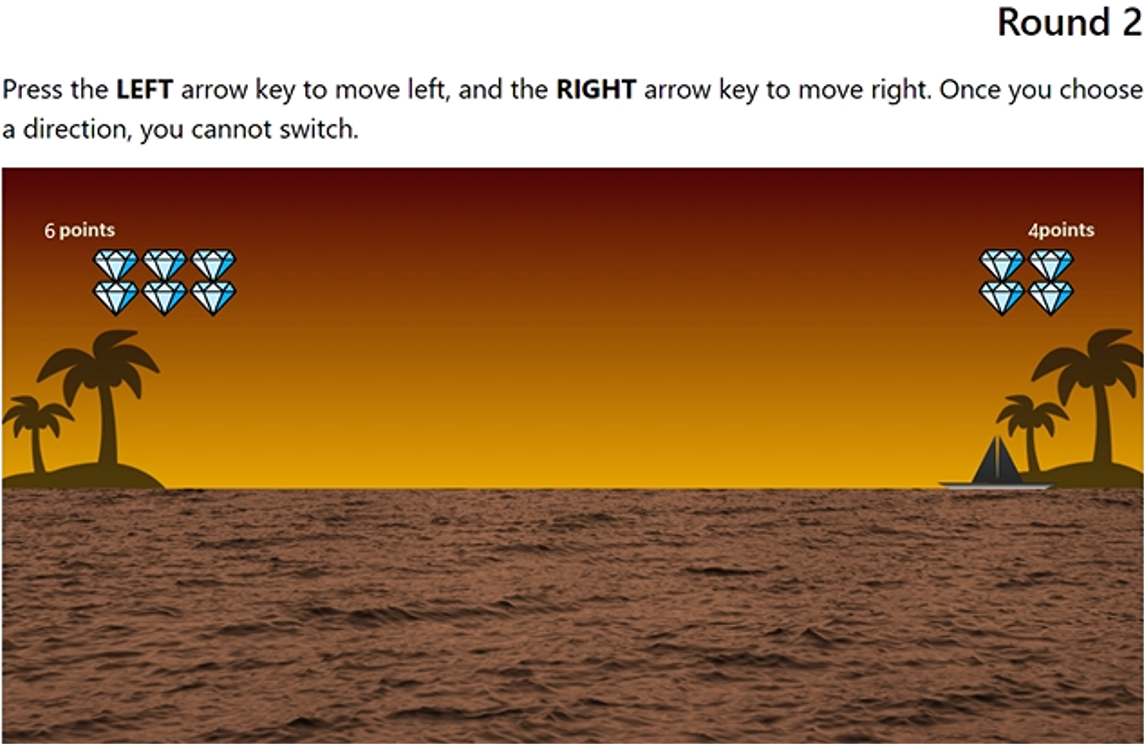 |

| **Table S1**  *Trials in the Boat task* | | | | | | | |
| --- | --- | --- | --- | --- | --- | --- | --- |
| Round | Payoff large | Payoff small | Starting point (% distance to large payoff) | Number of steps to large payoff | Number of steps to small payoff | Time until large island | Time until small island |
| 1 | 6 | 4 | 83% | 10 | 2 | 100s | 20s |
| 2 | 6 | 4 | 92% | 11 | 1 | 110s | 10s |
| 3 (catch) | 6 | 2 | 25% | 3 | 9 | 30s | 90s |
| 4 | 8 | 4 | 83% | 10 | 2 | 100s | 20s |
| 5 | 8 | 4 | 75% | 9 | 3 | 90s | 30s |
| 6 | 6 | 4 | 92% | 11 | 1 | 110s | 10s |
| 7 | 6 | 4 | 75% | 9 | 3 | 90s | 30s |
| 8 | 8 | 4 | 92% | 11 | 1 | 110s | 10s |
| 9 | 8 | 4 | 83% | 10 | 2 | 100s | 20s |
| 10 | 8 | 4 | 92% | 11 | 1 | 110s | 10s |
| 11 | 6 | 4 | 75% | 9 | 3 | 90s | 30s |
| 12 | 6 | 4 | 83% | 10 | 2 | 100s | 20s |
| 13 | 8 | 4 | 75% | 9 | 3 | 90s | 30s |

The outcome measure was the proportion of regular rounds in which participants chose to travel to the further away island (i.e., waiting longer to obtain the large reward).

**Dropout criterion:** Participants were excluded if they chose the smaller and farther reward in the catch round.

## Risk

### Balloon Analogue Risk Task (Risk_Bart)

The Balloon Analogue Risk Task (BART) is a computerized behavioral measure for risk taking^16^. In a recent large-scale evaluation of different risk measures, the BART was the behavioral measure that correlated most with propensity and frequency risk measures that are usually assessed via verbal reports^17^. In this task, participants can pump up a series of balloons by clicking on a button on the screen. Before each click, they can decide if they want to pump up the balloon further or collect the reward of the current value of the balloon (see Figure S5). The bigger the balloon, the bigger the reward they can collect. However, the balloon will pop at some point and in this case, participants get nothing for the current balloon.

| **Figure S5**  *Screenshot of the Balloon Analogue Risk Task.* |
| --- |
| 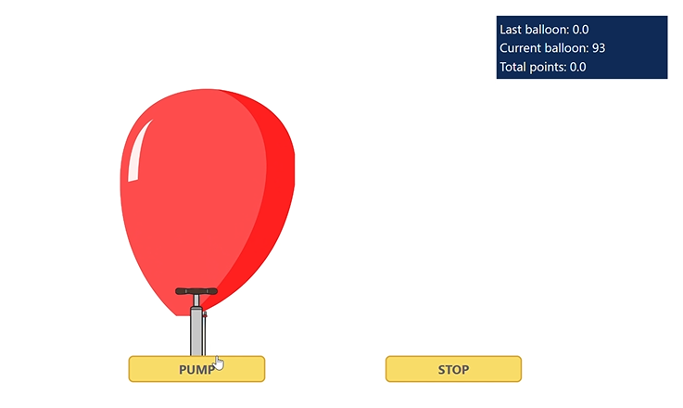 |
| *Note.* Screenshot of a balloon currently worth 93 points. The previous round didn’t yield any points (Last balloon: 0.0). This participant has so far collected no points (Total points: 0.0). |

Participants got to pump up twenty balloons. The maximum number of pumps per balloon was 128. We calculated breakpoints such that for every block of ten trials the average breakpoint was 64 pumps (see also Lejuez et al.^16^). Each pump increased the current balloon value by one point. Once collected, this value was added to the participants’ bank. If a balloon popped, participants got no money for this balloon, but the money in the bank remained untouched. Our outcome measure was the average adjusted number of pumps (defined as the average number of pumps excluding balloons that exploded). Adjusted number of pumps is preferable to total number of pumps, because the number of pumps is necessarily constrained on balloons that exploded, thereby limiting between-subjects variability in the absolute averages.

**Dropout criterion:** Participants were excluded if they pumped up all balloons until they exploded or never pumped any balloons to finish the experiment faster.

### Experienced-based risky choice task (Risk_Exp)

In this task, participants acquire information about the riskiness of choice options by experiencing the outcomes. This stands in contrast to description-based risky choice tasks, in which participants are explicitly informed about the reward probability of an option (see below). The information format (experienced vs described) has been shown to matter for people’s risk preferences in a number of studies^17–19^.

In this experience-based risky choice task, participants could repeatedly sample from the options on the screen and learn about the outcomes that are associated with each of them (see Figure S6). Outcomes were determined by random draws from the pre-defined distribution underlying each particular option. Each pair consisted of a riskier and a less risky option. Participants saw three pairs of doors; each pair was presented fifty times in a row. Each door differed in which payoff it hid and the underlying distribution according to which the payoff was determined. We only included positive payoffs and outcome probabilities of the risky options were always 0.5. In each round, participants had to click on a door and received the payoff behind that door. They also saw what was hidden behind the other door–the one they did not choose (i.e., a full-feedback protocol). Door location was counterbalanced such that each door appeared equally often on the left and right side. The following three choice pairs were used in the test battery:

- Pair 1: Option A = 6 or 0 points with a 50/50 chance, Option B = 3 points for sure
- Pair 2 (catch round): Option A = 2 or 4 points with a 50/50 chance, Option B = 0 points for sure
- Pair 3: Option A = 7 or 1 points with a 50/50 chance, Option B = 4 points for sure

Note that Pair 2 is a catch round to check if participants pay attention to the task. Our outcome measure is the proportion of risky choices in Pairs 1 and 3.

| **Figure S6**  *Screenshots from the Risk-from-Experience task.* | |
| --- | --- |
| 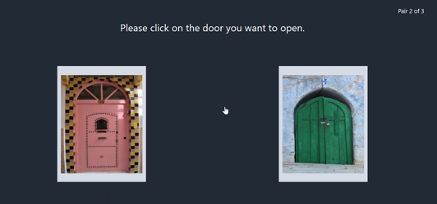 | 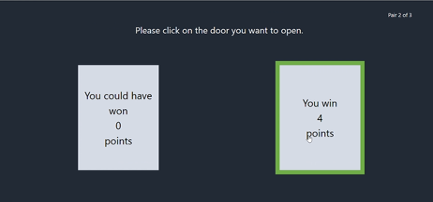 |

**Dropout criterion**: Participants were excluded if they picked the strictly worse option more than 60% of the time in the catch round.

### Description-based risky choice task (Risk_Desc)

We presented participants with ten lottery choice decisions with low payoffs as per Holt and Laury^20^. Participants had to indicate which monetary choice option they preferred. Each pair consisted of a riskier and a less risky option (Table S2). Option B (depicted on the right) was considered the riskier option because the potential payoffs were more variable. Our outcome measure was the proportion of risky choices across all ten trials.

| **Table S2**  *Choices in Risk_Desc.* | | |
| --- | --- | --- |
| Trial | Option A | Option B |
| 1 | 10 % chance to win £2.00  90 % chance to win £1.60 | 10 % chance to win £3.85  90 % chance to win £0.10 |
| 2 | 20 % chance to win £2.00  80 % chance to win £1.60 | 20 % chance to win £3.85  80 % chance to win £0.10 |
| 3 | 30 % chance to win £2.00  70 % chance to win £1.60 | 30 % chance to win £3.85  70 % chance to win £0.10 |
| 4 | 40 % chance to win £2.00  60 % chance to win £1.60 | 40 % chance to win £3.85  60 % chance to win £0.10 |
| 5 | 50 % chance to win £2.00  50 % chance to win £1.60 | 50 % chance to win £3.85  50 % chance to win £0.10 |
| 6 | 60 % chance to win £2.00  40 % chance to win £1.60 | 60 % chance to win £3.85  40 % chance to win £0.10 |
| 7 | 70 % chance to win £2.00  30 % chance to win £1.60 | 70 % chance to win £3.85  30 % chance to win £0.10 |
| 8 | 80 % chance to win £2.00  20 % chance to win £1.60 | 80 % chance to win £3.85  20 % chance to win £0.10 |
| 9 | 90 % chance to win £2.00  10 % chance to win £1.60 | 90 % chance to win £3.85  10 % chance to win £0.10 |
| Catch trial | 100 % chance to win £2.00  0 % chance to win £1.60 | 100 % chance to win £3.85  0 % chance to win £0.10 |

**Dropout criterion:** Participants were excluded if they still picked Option A in the last trial, which was a strictly dominated choice.

## Reciprocity measures

### Zürich Prosocial Game (Coop_Zurich)

The task is a modified version of the Zürich Prosocial Game^21^. In this game, participants try to reach a treasure by navigating a smiley-character along a path. Doors can unexpectedly appear and block the way; participants can open the doors if they have a key with matching color. Another player is visible on the playing field, but participants are instructed that others use the same field but play their own games. Although players pursue their goals independently, they can help each other out with keys if they want.

Previous studies found that helping in the Zürich Prosocial Game correlated with behaviour in the Dictator game^21^, that Zürich Prosocial Game game type variations (reciprocity, cost) overall load on different factors of prosociality^22^, and that Social Value Orientation predicted helping in the Zürich Prosocial Game^23^.

For this study, we developed new trial types that tapped into prospective reciprocity rather than retrospective reciprocity as in the original Zürich Prosocial Game. Participants received two trial types, reciprocity and baseline, in a blocked design (6 trials of each type). Participants were informed that they would be paired with a different player in each game. To add credibility to the game, before every trial, a mock pairing screen was presented, which appeared to be searching for another player. However, in reality the other player was not a real person, but an algorithm that used a Tit-for-Tat strategy during reciprocity trials. On reciprocity trials, the other player needed help and was potentially able to reciprocate helping later in the game (they had a key of the other color left). The participant could help at a cost: by helping the other player, they risked losing a key that they could potentially need for themselves later in the game. In all six reciprocity trials, the other player always played tit-for-tat, helping back if the participant helped and not helping if the participant did not help. Nevertheless, in two trials, even if the participant had helped, they could not reach the treasure because a door fell on their way, and the participant had no keys left. On baseline control trials, the participant could help at a cost, but it was apparent that the other player would not be able to reciprocate later in the game (because they had no keys left). In the baseline trials, if participants helped, they could not reach the treasure at the end, because they had no more keys left to unblock the path for themselves.

In each trial, both players had 60 seconds to reach their treasure. If they did not reach their treasure in time or were stuck behind a door for seven seconds, the smiley avatar “died” and the player did not receive any reward for this round. Both players were endowed with one blue key and one red key. Keys could only be used once and disappeared after use. They could be used to open doors that blocked the participant’s path or the other player’s path. After a player died, they could still help the other player out with their keys. However, when a player had, reached their treasure, they could no longer use their remaining keys to open doors for the other player. Figure S8 shows a screenshot of the playing field. Prior to the test trials, participants played practice rounds to learn to navigate the game. We also asked them comprehension questions to ensure they understood the information in all displayed elements of the playing field. The outcome measure was the difference between probability to help in reciprocity trials minus probability to help in baseline trials.

| **Figure S7**  *Screenshot of the Zürich Prosocial Game.* |
| --- |
| 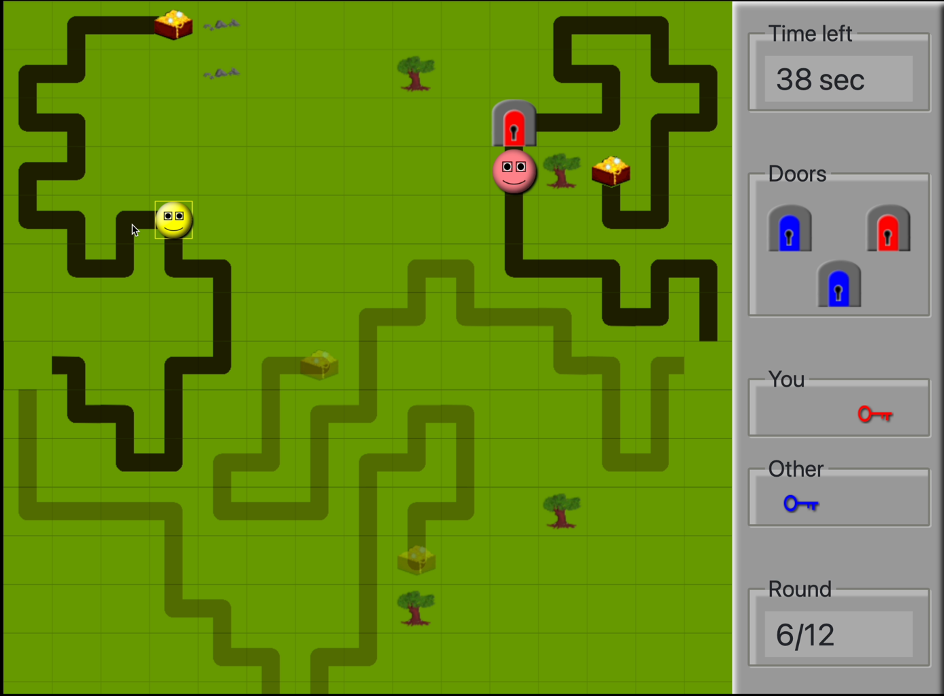 |
| *Note.* In this screen shot, the players have 38 seconds left to reach their treasures. Two blue doors and one red door will be placed on the playing field in the remainder of this round. The participant still has a red key, and the other player still has a blue key. The other player (pink smiley) is currently blocked by a red door, which they cannot open themselves. The participant (yellow smiley with square frame) can use their red key to open the door for the other player. |

**Dropout criterion:** Participants were excluded if they answered more than two of the four comprehension questions incorrectly or when they played less than three trials per condition (i.e., produced no time-outs in at least 3 trials per condition).

### Centipede game (Coop_Cent)

The Centipede game is played with two players who take alternating turns^24–26^. Similar to the image of a centipede with its many body segments, the players make their way through the long body of the game and can decide at each node if they want to continue to the next segment or stop the game (see Figure S7). Prior to the first decision, both players received complete information about the payoffs at each node of the task, including the final payoff they would receive if they make it through the whole body of the game. If Player 1 cooperated, the game moved to the next decision node and Player 2 decided to cooperate or stop. If Player 2 also continued, the game moved to the next decision node and it was Player 1’s turn again, and so on. Through mutual cooperation until the final stage of the game, the players could each reach a final payoff that exceeded their individual payoffs at the start of the game. On any given node, however, if one player defected and stopped the game, the other received less than they would have received in the previous round.

In the current Centipede task, participants always played the role of Player 1. Player 2 was not a real person. During practice rounds, participants experienced that other players stop the game before reaching the final node (also during practice rounds, other players were not real players but a computer program). However, in the test game, “Player 2” was pre-programmed to always cooperate such that participants could achieve the highest payoff by initiating and maintaining tit-for-tat reciprocity. To find more individual variation in the participants’ strategies, we presented a test game with 20 decision nodes, in which there was substantial inequity between the two players’ payoffs at each node, and where reaching the final end point was less profitable than other previous nodes because there was a drop in payoffs at the very end (even though they were still higher than at the start). At each node, we gave participants a window of 15 seconds to make their decision. If the participant was too slow to respond, they were timed-out and did not earn any points. The measure of interest was the number of cooperative decisions.

| **Figure S7**  **S8**  *Screenshot of the final nodes (16-20) of the test game in the Centipede game.* |
| --- |
| 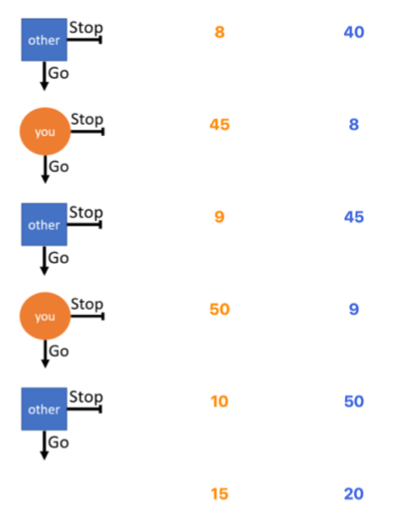 |
| *Note.* Player 1 (the participant) payoffs at each node are depicted in orange; Player 2 payoffs are depicted in blue. |

**Dropout criterion:** Participants were excluded if they did not make a choice in the test game.

### Trust/Dictator game (Coop_Trust)

The **Trust game** is an established and extensively used experimental measure of trust in economics and psychology^27,28^. There are two players: the “investor” is given a monetary endowment and can choose to send some of it to another player, the “trustee”. The amount sent is multiplied by some factor (often by 3) before it reaches the trustee. The trustee can then send money back to return the favor, but there is no guarantee that they will. Social pressure is minimal because the procedure is fully anonymous. There are numerous versions of the Trust game that differ in the amount endowed to the players, by how much the investment is multiplied, whether the investment decision is continuous (send any amount) or between predefined categories, how many rounds are played, whether there are role switches and many more (for an overview, see^29,30^). The Dictator game is similar to the Trust game but without the possibility for the second player to send money back. The first player, the “dictator” is the only one who gets to decide anything, namely the amount they want to send to the recipient. The Trust game has an element of prospective reciprocity, but general prosocial preference may also influence how much the first player invests. It has been suggested that a combination of Trust game and Dictator game can help to control for confounds of prosocial preferences in participants behavior in the Trust game^29,31,32^. Therefore, we presented both a Trust game and a Dictator game to our participants and used a difference score to assess how much of the investor’s behaviour in the Trust game can be attributed to their prosocial preferences. Participants first played the Trust Game, then the Dictator game. They were told that they would be paired with another player anonymously each time and that these were one-shot games (i.e., they only play once). Once they were allegedly paired with another player, they were instructed about the rules of the game and then got to make their decision on how much money to send. In both games, participants were assigned the role of the first mover (investor and dictator, respectively) and were endowed with £5. We were only interested in the investor’s behavior. In our Trust game, the amount sent was multiplied by three, and the trustee was not a real person. Instead, unbeknownst to the participants, the pre-programmed response was to send back half of the amount in the trustee’s possession. We were interested in individual differences in a one-shot scenario for each game. Therefore, we did not restrict participants’ decisions to categorical choices but let them send any amount between 0 and £5 to have a continuous measure of trusted amount. Our outcome measure for this task was the difference score between donations in the two games (donation amount in Trust game minus donation in Dictator game). We asked three comprehension questions, before participants made a choice in the Trust game. For each question, we presented four possible answers of which only one was correct.

Question 1: Imagine you send £3.00 to the other player. What is the range of possible values that the other can send back to you?

- - £0 - £3
  - £0 - £9 (correct)
  - £3 - £9
  - £3 - £12

Question 2: Imagine you send £3.00 to the other player and the other player sends back £3.00 to you. How much do you have at the end?

- - £2
  - £5 (correct)
  - £6
  - £12

Question 3: Imagine you send £0.00 to the other player. What is the range of possible values that the other can send back to you?

- - £0 - £0 (correct)
  - £0 - £5
  - £5 - £10
  - £5 - £15

**Dropout criterion:** Participants were excluded if they answered more than one question incorrectly.

# Participants and Procedure

A session consisted of three or four tasks (see Table 1 for the composition of each session). Upon clicking the Study link, participants received general information about the experiment, including a consent form where they agreed with the particular conditions of this experiment: namely, that they agreed to participate in all three sessions and were aware that they would only receive the bonus payment if they completed all three sessions. Participants were also informed that we have interspersed catch trials and questions to make sure they conduct the task with sufficient attention and that failing these attention checks can lead to not being invited to the next part of the experiment. In this case, they would receive their participation fee but no bonus payment. Furthermore, participants were informed that the sessions would only be accessible at certain times: specifically, Session 1 was accessible immediately, Session 2 would be accessible on the next day, and Session 3 on the day after that. We chose to limit the time slots to three hours so that participants would treat the subsequent parts of the experiment as sincere appointments. In addition, this constraint aimed to increase credibility of being paired with a social partner for the reciprocity tasks, because many other participants were performing the test battery at the same time. After data collection for each session was closed, we customized a Prolific “Allow list” for the next session, which specified the IDs of those participants who finished the previous session successfully (i.e., participants who have returned the study to Prolific in time and who were not identified by us as dropouts due to failure in the attention checks; for details on attention checks, see supplementary information on each task). Based on this “Allow list”, we opened the next study session for the specified time on the following day. This procedure was repeated after Session 2. After Session 3 was closed, we assessed how many participants had successfully completed the test battery. Figure S9 provides a Flow chart of participant recruitments and exclusions.

| **Figure S9**  *Flow chart of participant recruitments and exclusions.* |
| --- |
|  |
| *Note.* Data was collected in three waves; each wave entailed three sessions during which the ten tasks were presented. The diagram shows the number of recruited participants and how many participants were excluded in each step. Some participants failed more than one inclusion criterion, and some participants passed our criteria, but nevertheless chose to not finish the experiment. |

# Additional information on analysis and results

## General approach

We detailed the initial analysis plan in a pre-registration, where we planned to use Structural Equation Modelling (Plan A), or multivariate multiple regression (Plan B) in case the obtained data was unsuitable for the former (https://osf.io/prm5g).

The pattern of correlations observed in the data among the three measures of strategic future-oriented reciprocal cooperation (Coop_Trust, Coop_Cent, Coop_Zurich) and the different indicators of the three cognitive variables (Plan_Tower, Pat_Hybrid, Pat_Kirby, Pat_Boat, Risk_Bart, Risk_Exp, Risk_Desc) ranged merely from low to moderate (see Table S3). This finding led us to discard the initially proposed approach of using Structural Equation Modeling (SEM) because SEM requires a substantial interdependence among the variables to accurately model their relationships and capture latent constructs effectively. With weak correlations, the assumption of meaningful associations required for SEM was not met, making it not appropriate for our data. Instead, three multiple regressions were conducted, one for each of the measures of prospective reciprocity as the dependent variable.

Initially, the implementation of a multivariate multiple regression model was proposed for the analysis, since it was expected that the social variables would be correlated. However, the absence of statistically significant correlations among the outcome variables that constituted the prospective reciprocity construct steered the analysis towards the use of three separate multiple regression models. This decision was based on avoiding assumptions of interactions between the dependent variables, which multivariate multiple regression assumes, even when such interactions may not be present or significant. Employing separate univariate models guarantees a more precise representation of the relationships between variables. In the multiple regression models, a bootstrap with 1000 replacements was used to obtain more accurate estimates of the standard errors and confidence intervals of the coefficients. The bootstrapped standard errors and confidence intervals were calculated from the distribution of the bootstrap coefficients. The analyses were performed using IBM SPSS (version 29).

## Coefficients

In addition to *unstandardised* and *standardised coefficients*, *structure coefficients*, *Johnson's relative importance weights* (RIW)^33,34^ and the *relative importance* (in %) of the predictors based on RIW were calculated. *Standardised coefficients* (beta weights) are context dependent and may not be optimal for explanatory purposes, especially when dealing with highly correlated predictors. Despite the absence of significant multicollinearity problems, in our specific case, the inclusion of additional coefficients provides a more accurate representation of the relationship between the predictors and the criterion variable. This approach is in line with the theoretical and explanatory goals of our model. The *structure coefficients* represent the correlation between each predictor and the estimated score. A squared structure coefficient indicates how much variance the predictor can explain of the observed *R*^2^ effect, providing insight into the potential contribution of each predictor to the overall model^35^. Unlike *beta weights*, *structure coefficients* provide information about the bivariate relationship independent of other predictors, making them valuable in the presence of multicollinearity. They also help to identify *suppressor predictors*, where a predictor may have a substantial beta weight but a small structure coefficient^35^. A suppressor variable is a variable that does not have a significant direct relationship with the dependent variable, but including it in the model enhances the predictive power of other variables. *RIW*s estimate the influence of each variable in predicting the criterion variable, taking into account both individual and combined contributions. In other words, *RIW*s provide an additional level of insight into the individual impact of each predictor while also considering their combined effects^33^. The percentage of relative importance measures the proportional contribution of each predictor to *R*^2^. It indicates the percentage of *R*^2^ that a given predictor can predict, taking into account the influence of intercorrelations between predictors. The analyses was performed using ﻿MIMR-Corr.sps^36^.

## Zero-inflated Beta regression (ZOIBR)

For the Centipede game, we also employed a zero-one-inflated beta regression (ZOIBR) model^37^, implemented within a Bayesian framework using the brms R package^38^. The ZOIBR model was chosen due to the prevalence of 0s and 1s in the scores representing the number of cooperative decisions (0 and 1 in proportions), leading to a tri-modal distribution (one mode for participants who never cooperate, another for those who always cooperate, and a unimodal distribution for the remaining participants, as illustrated in Figure 2 in the manuscript). The ZOIBR model comprises two components: a beta distribution for responses within the closed (0, 1) interval and a Bernoulli distribution for binary {0, 1} responses. In this model, predictors may influence either or both the continuous and binary responses, the proportion of binary responses, or the spread of the continuous proportions.

The model, which can be conceptualized as a mixture of beta and logistic regressions, is described by four parameters: α, γ, μ and φ. Parameter α represents the probability that an observation is either 0 or 1. If an observation is 0 or 1, the conditional probability of it being 1 is given by γ. If an observation is neither 0 nor 1, it is described by the beta distribution with a mean μ and precision φ. This model enables separate tests on whether the seven variables act as predictors for the mean (μ) and precision (φ) of the beta distribution of continuous cooperative decisions (0, 1), the zero-one inflation probability α, indicating the probability of extreme responses (never cooperate/always cooperate), and the conditional one-inflation probability γ, given an extreme response, the probability of the response being always cooperate. For the analyses, the number of cooperative decisions was transformed into proportions. The Bayesian pseudo-*R*^2^ was estimated for the whole model.

## Correction for attenuation

In structural equation modelling (SEM), parameter estimates are adjusted for attenuation due to measurement error, so they tend to be larger than those obtained by multiple regression (Hair et al., 2019). In correlation and regression analyses, unreliable measurements can result in underestimated relationships and elevate the risk of Type II errors. This issue is especially relevant when the research objective involves capturing the inherent relationships present within the population with precision. While reliability estimates (such as Cronbach's alphas) ranging from 0.7 to 0.8 are acceptable^39^, it's noteworthy that measurements of even this reliability encompass enough measurement error to necessitate correction. Although the concept of correction for attenuation addressing low reliability is thoroughly discussed in numerous regression texts^39–44^, its practical implementation in academic literature is not widespread. Here, we replicate the multiple regression analyses incorporating a correction for attenuation to achieve a more accurate representation of the true relationships between variables.

We apply

$$r_{xy}^{*}= \frac{r_{xy}}{\sqrt{r_{xx'}*r_{yy'}}}$$

Where:

r_xy_ is the observed correlation between variables x and y.

$r_{xx'}$ is the reliability of variable x.

$r_{yy'}$ is the reliability of variable y.

Internal consistency reliability was estimated for those tasks that met the necessary conditions (had different trials available to apply the procedure). In case of Coop_Trust and Coop_Cent scores, where the tasks did not meet these conditions, it was not possible to compute reliability estimates. Reliability coefficients were estimated by the split-half method, using the scores of the even and odd items. Spearman-Brown coefficients were calculated for Coop_Zurich, Risk_Exp, Risk_Bart, Risk_Dec, Pat_Kirby, and Pat_Boat, and intraclass correlation coefficient (ICC) for Pat_Hybrid.

## Results

### Correlations and reliability coefficients

Table S3 presents the observed correlation coefficients between outcome variables and predictors, along with the reliability coefficients. The split-half reliability analysis yielded varying results across the different measures. The Pat_Kirby and Pat_Boat measures demonstrated high levels of internal consistency, with Spearman-Brown Coefficients of .95 and .92, respectively. These findings suggest that Coop_Zurich, Risk_Desc, Pat_Kirby and Pat_Boat measures are highly reliable, while caution is warranted when interpreting results from the Risk_Bart and Pat_Hybrid measures due to their low internal consistency.

| **Table S3**  *Means, standard deviations, Pearson correlation coefficients, and reliability estimations*. | | | | | | | | | | | | |
| --- | --- | --- | --- | --- | --- | --- | --- | --- | --- | --- | --- | --- |
|  | *M* | *SD* | 1 | 2 | 3 | 4 | 5 | 6 | 7 | 8 | 9 | 10 |
| 1. Coop_Trust | .24 | 1.45 |  |  |  |  |  |  |  |  |  |  |
| 2. Coop_Cent | 5.31 | 3.58 | .03 |  |  |  |  |  |  |  |  |  |
| 3. Coop_Zurich | .34 | .31 | .06 | .03 | ***.74*** |  |  |  |  |  |  |  |
| 4. Risk_Exp | .36 | .14 | **-.14^*^** | -.05 | **-.14^*^** | ***.80*** |  |  |  |  |  |  |
| 5. Risk_Bart | 46.7 | 18.04 | .08 | **.20^**^** | **.23^**^** | -.07 | ***.33*** |  |  |  |  |  |
| 6. Risk_Desc | .38 | .19 | -.09 | .02 | -.01 | .09 | -.01 | .***87*** |  |  |  |  |
| 7. Pat_Hybrid | 15.60 | 10.38 | .07 | .**16^**^** | **.18^**^** | -.09 | **.32^**^** | -.05 | ***.50*** |  |  |  |
| 8. Pat_Kirby | .49 | .20 | **.15^*^** | .03 | -.01 | -.07 | **.16^**^** | -.02 | **.27^**^** | ***.95*** |  |  |
| 9. Pat_Boat | .40 | .37 | -.02 | .02 | **.11^*^** | -.03 | **.22^**^** | -.05 | **.45^**^** | **.27^**^** | ***.92*** |  |
| 10. Plan_Tower | 7.79 | 1.05 | -.09 | -.01 | .06 | **.14^*^** | **-.21^**^** | .08 | -.11 | **-.21^**^** | .00 | ***.67*** |
| *Note.* Single and double asterisks indicate statistical significance at .95 and .99 level, respectively. | | | | | | | | | | | | |

### Multiple regressions

In addition to the results presented in the main manuscript, we assessed the importance of predictor variables using additional coefficients, and conducted a multiple regression analysis on the disattenuated correlation matrix based on the estimated internal consistency reliability coefficients of both the independent variable and the predictors.

#### Zürich Prosocial Game

##### Coefficients

In Table S4, we present *standardized coefficients* (beta weights), *structure coefficients*, Johnson's *relative importance weights* and the *relative importance* (in %) of predictors, based on Johnson's relative weights. Table S5 shows the ranking order based on the different coefficients. Based on *RIW*, Risk_Bart would be considered the most important variable (RI = 45.2%), followed by Pat_Hybrid (RI = 20.5%) and Risk_Exp (RI = 17.6%). Results also show that Plan_Tower could have a suppressor effect: its correlation with the Coop_Zurich is not statistically significant, but when introduced into the model, the weight becomes statistically significant. Pat_Boat, on the other hand, which had a statistically significant correlation, when introduced into the model, ceases to be significant, due to shared variance with other variables in the model; note that we see its structure coefficient is higher than Plan_Tower (0.36 compared to 0.18).

| **Table S4**  *Multiple regression coefficients for predicting Coop_Zurich score from all cognitive measures using bootstrap (N = 1000): standardized coefficients (beta), structure coefficients, relative importance weights and the relative importance (in %).* | | | | |
| --- | --- | --- | --- | --- |
| **Predictors** | **beta** | **Structure** | **RIW** | **RI(%)** |
| Risk_Bart | 0.214 | 0.730 | 0.217 | 45.2 |
| Patience_Hybrid | 0.122 | 0.572 | 0.146 | 20.5 |
| Risk_Exp | -0.133 | -0.434 | -0.134 | 17.6 |
| Plan_Tower | 0.118 | 0.180 | 0.089 | 8.3 |
| Pat_Boat | 0.027 | 0.360 | 0.068 | 5.7 |
| Pat_Kirby | -0.071 | -0.038 | -0.047 | 2.7 |
| Risk_Desc | 0.005 | -0.02 | 0.00 | 0.1 |

| **Table S5**  *Ranking of importance based on different coefficients.* | | |
| --- | --- | --- |
| **Beta Order** | **Structure Order** | **Relative Order** |
| 1. Risk_Bart | 1. Risk_Bart | 1. Risk_Bart |
| 2. Risk_Exp | 2. Pat_Hybrid | 2. Pat_Hybrid |
| 3. Pat_Hybrid | 3. Risk_Exp | 3. Risk_Exp |
| 4. Plan_Tower | 4. Pat_Boat | 4. Plan_Tower |
| 5. Pat_Kirby | 5. Plan_Tower | 5. Pat_Boat |
| 6. Pat_Boat | 6. Pat_Kirby | 6. Pat_Kirby |
| 7. Risk_Desc | 7. Risk_Desc | 7. Risk_Desc |

##### Multiple regression corrected for attenuation (for both Coop_Zurich and predictors)

Multiple regression analysis was then conducted on the disattenuated correlation matrix based on the estimated internal consistency reliability coefficients of both the independent variable and the predictors (Table S6). The new regression model accounts for a significant amount of variance in the dependent variable, Coop_Zurich (*R*² = .181, adjusted *R*² = .160). The overall model is statistically significant (*F*(7, 271) = 8.54, *p* < .001). Unstandardized and standardized coefficients estimations are presented in Table S7.

| **Table S6**  *Disattenuated correlations between Coop_Zurich and predictors corrected for reliability coefficients* | | | | | | | |
| --- | --- | --- | --- | --- | --- | --- | --- |
|  | 3 | 4 | 5 | 6 | 7 | 8 | 9 |
| 3. Coop_Zurich |  |  |  |  |  |  |  |
| 4. Risk_Exp | -.18 |  |  |  |  |  |  |
| 5. Risk_Bart | .31 | -.09 |  |  |  |  |  |
| 6. Risk_Desc | -.01 | .11 | -.01 |  |  |  |  |
| 7. Pat_Hybrid | .25 | -.12 | .42 | -.07 |  |  |  |
| 8. Pat_Kirby | -.01 | -.09 | .21 | -.03 | .35 |  |  |
| 9. Pat_Boat | .15 | -.04 | .30 | -.07 | .59 | .32 |  |
| 10. Plan_Tower | .10 | .16 | -.21 | .08 | -.11 | -.21 | .00 |

| **Table S7**  *Regression coefficients from correlation matrix corrected for attenuation (for Coop_Zurich and predictors). Bold text indicates statistically significant coefficients.* | | | | | |
| --- | --- | --- | --- | --- | --- |
|  | Unstandardized Coefficients | | Standardized Coefficients | *t* | Sig. (2-tailed) |
|  | B | Std. Error | Beta |  |  |
| (Constant) | -.173 | .164 |  | -1.08 | .28 |
| **Risk_Exp** | **-.388** | **.125** | **-.173** | **-3.08** | **<0.01** |
| **Risk_Bart** | **.005** | **.001** | **.286** | **4.61** | **<0.01** |
| Risk_Desc | .005 | .091 | .005 | .10 | .92 |
| **Pat_Hybrid** | **.005** | **.002** | **.173** | **2.35** | **.02** |
| Pat_Kirby | -.164 | .094 | -.104 | -1.71 | .09 |
| Pat_Boat | -.009 | .058 | -.011 | -.16 | .88 |
| **Plan_Tower** | **.055** | **.017** | **.185** | **3.17** | **<0.01** |

##### Multiple regression corrected for attenuation (in predictors’ reliability)

Multiple regression analysis was conducted on the disattenuated correlation matrix based on the estimated internal consistency reliability coefficients of the predictor variables. The correlation matrix in Table S8 shows the relationships between Coop_Zurich and all predictor variables, corrected for attenuation.

| **Table S8**  *Disattenuated correlations between Coop_Zurich and predictors corrected for predictors reliability coefficients* | | | | | | | |
| --- | --- | --- | --- | --- | --- | --- | --- |
|  | 3 | 4 | 5 | 6 | 7 | 8 | 9 |
| 3. Coop_Zurich |  |  |  |  |  |  |  |
| 4. Risk_Exp | -.154 |  |  |  |  |  |  |
| 5. Risk_Bart | .259 | -.09 |  |  |  |  |  |
| 6. Risk_Desc | -.007 | .107 | -.015 |  |  |  |  |
| 7. Pat_Hybrid | .235 | -.127 | .456 | -.075 |  |  |  |
| 8. Pat_Kirby | -.012 | -.085 | .185 | -.027 | .351 |  |  |
| 9. Pat_Boat | .119 | -.032 | .258 | -.05 | .602 | .283 |  |
| 10. Plan_Tower | .070 | .192 | -.284 | .105 | -.173 | -.265 | .004 |

The multiple R-squared of this model increased compared to the previous one (*R*^2^ = .137, 95 % bootstrap *CI* [.086, .279]), indicating that approximately 13.7 % of the variance in the Coop_Zurich score can be explained by the predictor variables.

Table S9 shows unstandardized and standardized coefficient estimations, bootstrap 95 % Confidence Interval for B and the relative contribution to the multiple R, indicating the percentage of variance in the dependent variable that can be attributed to each predictor variable. Measures of relative importance are provided based on Johnson’s relative weight procedure^33,34^, which measures the proportionate contribution each predictor makes to *R*^2^ (i.e., the relative importance) after correcting for the effects of the intercorrelations among predictors.

| **Table S9**  *Regression coefficients from correlation matrix corrected for attenuation for the Coop_Zurich model and relative contribution to multiple R (reported as percentages) point-estimate and bootstrap 95 % confidence interval. Bold text indicates statistically significant coefficients.* | | | | | | | |
| --- | --- | --- | --- | --- | --- | --- | --- |
|  | Coefficients | | Bootstrap 95 % Confidence Interval for B | | Relative Contribution to Multiple R (Percentages) Point-Estimate and Bootstrap 95 % | | |
|  | B | beta | Lower Bound | Upper Bound | Point- Estimate | Lower  Bound | Upper  Bound |
| **Risk_Exp** | **-.360** | **-.156** | **-.629** | **-.086** | **20.1** | **1.3** | **43.7** |
| **Risk_Bart** | **.004** | **.226** | **.002** | **.006** | **42.1** | **13.0** | **61.8** |
| Risk_Desc | .009 | .003 | -.190 | .220 | .1 | .1 | 13.5 |
| **Pat_Hybrid** | **.004** | **.123** | **.000** | **.008** | **18.3** | **2.5** | **42.2** |
| Pat_Kirby | -.116 | -.061 | -.287 | .086 | 2.3 | .6 | 15.9 |
| Pat_Boat | .025 | -.022 | -.083 | .14 | 5.3 | 1.1 | 22.8 |
| **Plan_Tower** | **.051** | **.156** | **.001** | **.105** | **11.8** | **.9** | **39.6** |

#### Centipede Game

##### Multiple regression

Table S13 presents the results of the multiple regression analysis for the relationship between Coop_Cent, as the dependent variable, and the seven predictor variables. Two predictors showed statistically significant standardized coefficients. Specifically, riskier behavior (higher Risk_Bart scores) was associated with more cooperation in the Centipede game (*Beta* = .18, *p* < .01). Similarly, more patience (higher Pat_Hybrid scores) was associated with more cooperation (*Beta* = .15, *p* = .02). The remaining predictors were not statistically significant (all *p*-values < .05, see Table S13). Figure S10 visualizes the relationships of the two best predictors with the reciprocity task. The model accounted for 6.0 % of the variance in Centipede score ($R^{2}$= .060), with an *adjusted* $R^{2}$ of .037 ($F_{7, 289}$ = 1.97, *p* = .012). The Durbin-Watson statistic was 2.64, indicating no significant autocorrelation in the residuals. The remaining predictors did not reach statistical significance (all *p* values > .05).

| **Figure S10**  *Scatterplots and regression line for the two best predictor variables of the Centipede task.* | |
| --- | --- |
|  |  |

| **Table S11**  *Multiple regression coefficients for predicting Centipede score from all cognitive measures using bootstrap (N = 1000). Bold text indicates statistically significant coefficients.* | | | | | | | |
| --- | --- | --- | --- | --- | --- | --- | --- |
|  | Unstandardized Coefficients | | Standardized Coefficients | Bootstrap Std. Error | Bootstrap Sig. (2-tailed) | Bootstrap 95 % Confidence Interval for B | |
|  | B | Std. Error | Beta |  |  | Lower Bound | Upper Bound |
| (Constant) | 2.254 | 1.969 |  | 1,926 | .253 | -1.571 | 6.101 |
| Risk_Exp | -.791 | 1.512 | -.030 | 1.597 | .641 | -3.944 | 2.242 |
| **Risk_Bart** | **.036** | **.012** | **.180** | **.012** | **.008** | **.011** | **.058** |
| Risk_Desc | .462 | 1.072 | .025 | 1.071 | .672 | -1.506 | 2.656 |
| **Pat_Hybrid** | **.052** | **.023** | **.151** | **.024** | **.028** | .006 | .099 |
| Pat_Kirby | -.107 | 1.107 | -.006 | 1.085 | .923 | -2.259 | 2.010 |
| Pat_Boat | -.837 | .628 | -.087 | .621 | .179 | -2.111 | .392 |
| Plan_Tower | .140 | .204 | .041 | .205 | .509 | -.230 | .564 |

##### ZOIB Regression

Table S14 displays the recovered posterior means, standard deviations, and 95 % credible intervals for the ZOIBR model adjusted for Coop_Cent. Precision (phi Intercept) is modelled on the log scale. The other parameters are modelled in logit scale.

| **Table S12**  *Results for Coop_Cent using Zero-One-Inflated-Beta regression (ZOIBR). Regression coefficients (estimates), the standard error, and the lower and upper limit of the 95% credibility interval (CI). B*eta *distribution*: *estimate of predictors in* mean (μ) and precision (ϕ) of *the continuous process;* α*: estimates of the probability of a score being 0 or 1.* γ*: estimates of the probability of a cooperate being 1, given it is either 0 or 1. Bold text indicates when 0 not included in 95% CI.* | | | | |
| --- | --- | --- | --- | --- |
|  | **Estimate** | **Est. Error** | **95% CI Lower** | **95% CI Upper** |
| Coefficients for μ in beta regression model for continuous cooperative decisions | | | | |
| Intercept | -0.44 | 0.70 | -1.82 | 0.93 |
| Risk_Bart | 0.00 | 0.00 | -0.01 | 0.01 |
| Risk_Exp | -0.21 | 0.53 | -1.28 | 0.84 |
| Risk_Desc | 0.28 | 0.39 | -0.47 | 1.04 |
| **Pat_Hybrid** | **0.02** | **0.01** | **0.00** | **0.04** |
| Pat_Kirby | 0.18 | 0.38 | -0.56 | 0.93 |
| Pat_Boat | -0.17 | 0.23 | -0.61 | 0.27 |
| Plan_Tower | -0.03 | 0.07 | -0.16 | 0.11 |
| Coefficients for ϕ in beta regression model for continuous cooperative decisions | | | | |
| **Intercept** | **2.54** | **0.87** | **0.84** | **4.28** |
| Risk_Bart | -0.01 | 0.01 | -0.02 | 0.00 |
| Risk_Exp | 0.09 | 0.71 | -1.28 | 1.46 |
| Risk_Desc | -0.08 | 0.52 | -1.13 | 0.95 |
| Pat_Hybrid | 0.00 | 0.01 | -0.02 | 0.02 |
| Pat_Kirby | -0.70 | 0.52 | -1.70 | 0.33 |
| Pat_Boat | -0.01 | 0.28 | -0.57 | 0.53 |
| Plan_Tower | -0.11 | 0.08 | -0.27 | 0.04 |
| Coefficients for “probability of a score being 0 or 1 (not cooperate/always cooperate” (α) | | | | |
| Intercept | -2.41 | 1.29 | -5.03 | 0.09 |
| Risk_Bart | 0.01 | 0.01 | -0.01 | 0.02 |
| Risk_Exp | 1.72 | 1.01 | -0.26 | 3.67 |
| Risk_Desc | -0.19 | 0.70 | -1.60 | 1.16 |
| Pat_Hybrid | 0.00 | 0.01 | -0.03 | 0.03 |
| Pat_Kirby | 0.00 | 0.73 | -1.42 | 1.41 |
| Pat_Boat | -0.64 | 0.41 | -1.45 | 0.14 |
| Plan_Tower | 0.10 | 0.13 | -0.16 | 0.36 |
| Coefficients for “conditional probability of cooperate” (γ) | | | | |
| Intercept | -0.42 | 2.94 | -6.11 | 5.37 |
| **Risk_Bart** | **0.08** | **0.02** | **0.04** | **0.12** |
| Risk_Exp | -3.32 | 2.41 | -7.93 | 1.24 |
| Risk_Desc | 0.22 | 1.50 | -2.71 | 3.10 |
| Pat_Hybrid | -0.00 | 0.03 | -0.07 | 0.07 |
| Pat_Kirby | -1.82 | 1.53 | -4.83 | 1.24 |
| Pat_Boat | 0.09 | 0.93 | -1.71 | 1.88 |
| Plan_Tower | 0.04 | 0.33 | -0.60 | 0.69 |

#### Trust

##### Coefficients

In Table S10 we present *standardized coefficients* (beta weights), *structure coefficients*, Johnson's *relative importance weights* and the *relative importance* (in %) of predictors, based on Johnson's relative weights. Based on *RIW*, Pat_Kirby would be considered the most important variable (RI = 34.9%), followed by Risk_Exp (RI = 29.3 %). The same ranking for the predictor was found using all different coefficients.

| **Table S13**  *Multiple regression coefficients for predicting Coop_Trust from all cognitive measures using bootstrap (N = 1000): standardized coefficients (beta), structure coefficients, relative importance weights and the relative importance (in %).* | | | | |
| --- | --- | --- | --- | --- |
| **Predictors** | **beta** | **Structure** | **RIW** | **Rl (%)** |
| Pat_Kirby | .138 | .645 | .138 | 34.9 |
| Risk_Exp | -.114 | -.604 | -.124 | 29.3 |
| Risk_Desc | -.077 | -.398 | -.083 | 13.1 |
| Plan_Tower | -.028 | -.403 | -.059 | 7.1 |
| Risk_Bart | .056 | .329 | .057 | 6.2 |
| Pat_Boat | -.084 | -.067 | -.059 | 5.2 |
| Pat_Hybrid | .028 | .282 | .044 | 4.3 |

##### Multiple regression corrected for attenuation

Multiple regression analysis was then conducted on the disattenuated correlation matrix based on the estimated internal consistency reliability coefficients of the predictors (Table S11). The new regression model accounted for a significant amount of variance in the dependent variable Coop_Trust (*R*² = .06; (*CI*: .035; .149). The multiple R-squared estimation indicates that approximately 6 % of the variance in the dependent variable can be explained by the predictor variables. Table S12 shows unstandardized and standardized coefficient estimations, bootstrap 95 % confidence interval for *B,* and the relative contribution to the multiple *R*. Measures of relative importance are provided based on Johnson’s relative weight procedure.

| **Table S14**  *Disattenuated correlations between Coop_Trust score and predictors corrected for reliability coefficients.* | | | | | | | |
| --- | --- | --- | --- | --- | --- | --- | --- |
|  | 3 | 4 | 5 | 6 | 7 | 8 | 9 |
| 3. Coop_Zurich |  |  |  |  |  |  |  |
| 4. Risk_Exp | -0.16 |  |  |  |  |  |  |
| 5. Risk_Bart | 0.08 | -0.08 |  |  |  |  |  |
| 6. Risk_Desc | -0.10 | 0.11 | -0.01 |  |  |  |  |
| 7. Pat_Hybrid | 0.07 | -0.10 | 0.32 | -0.06 |  |  |  |
| 8. Pat_Kirby | 0.15 | -0.09 | 0.17 | -0.03 | 0.27 |  |  |
| 9. Pat_Boat | -0.02 | -0.03 | 0.23 | -0.05 | 0.47 | 0.28 |  |
| 10. Plan_Tower | -.11 | .19 | -.25 | .11 | -.13 | -.27 | .00 |

| **Table S15**  *Regression coefficients from correlation matrix corrected for attenuation for the Coop_Trust model and relative contribution to multiple R (reported as percentages) point-estimate and bootstrap 95 % confidence interval. Bold text indicates statistically significant coefficients.* | | | | | | | |
| --- | --- | --- | --- | --- | --- | --- | --- |
|  | Coefficients | | Bootstrap 95 % Confidence Interval for B | | Relative Contribution to Multiple R (Percentages) Point-Estimate and Bootstrap 95 % | | |
|  | B | beta | Lower Bound | Upper Bound | Point- Estimate | Lower  Bound | Upper  Bound |
| **Risk_Exp** | **-1.444** | **-.124** | **-2.839** | **-.153** | **32.1** | **2.1** | **59.4** |
| Risk_Bart | .004 | .048 | -.007 | .018 | 6.3 | .5 | 41.3 |
| Risk_Desc | -.580 | -.075 | -1.347 | .302 | 11.7 | .2 | 38.5 |
| Pat_Hybrid | .005 | .035 | -.018 | .027 | 25.50 | 5.20 | 48.90 |
| **Pat_Kirby** | **1.003** | **.135** | **.154** | **1.820** | **31.2** | **2.2** | **53.4** |
| Pat_Boat | -353 | -.089 | -.946 | .189 | 5.4 | .8 | 31.9 |
| Plan_Tower | -.056 | -.029 | -.286 | .173 | 9.2 | .8 | 42.9 |

# References

1. Berg, W. K. & Byrd, D. L. The Tower of London Spatial Problem-Solving Task: Enhancing Clinical and Research Implementation. *J. Clin. Exp. Neuropsychol.* **24**, 586–604 (2002).

2. Shallice, T. Specific impairments of planning. *Philos. Trans. R. Soc. Lond. B Biol. Sci.* **298**, 199–209 (1982).

3. Kaller, C. P., Rahm, B., Köstering, L. & Unterrainer, J. M. Reviewing the impact of problem structure on planning: A software tool for analyzing tower tasks. *Behav. Brain Res.* **216**, 1–8 (2011).

4. Kaller, C. P. *et al.* Assessing Planning Ability Across the Adult Life Span: Population-Representative and Age-Adjusted Reliability Estimates for the Tower of London (TOL-F). *Arch. Clin. Neuropsychol.* acv088 (2015) doi:10.1093/arclin/acv088.

5. Kaller, C. P., Unterrainer, J. M. & Stahl, C. Assessing planning ability with the Tower of London task: Psychometric properties of a structurally balanced problem set. *Psychol. Assess.* **24**, 46–53 (2012).

6. Unterrainer, J. M. *et al.* Assessing Planning Ability Across the Adult Life Span in a Large Population-Representative Sample: Reliability Estimates and Normative Data for the Tower of London (TOL-F) Task. *J. Int. Neuropsychol. Soc.* **25**, 520–529 (2019).

7. Kirby, K. N. One-year temporal stability of delay-discount rates. *Psychon. Bull. Rev.* **16**, 457–462 (2009).

8. Kirby, K. N. & Maraković, N. N. Delay-discounting probabilistic rewards: Rates decrease as amounts increase. *Psychon. Bull. Rev.* **3**, 100–104 (1996).

9. Kirby, K. N., Petry, N. M. & Bickel, W. K. Heroin addicts have higher discount rates for delayed rewards than non-drug-using controls. *J. Exp. Psychol. Gen.* **128**, 78–87 (1999).

10. Wileyto, E. P., Audrain-Mcgovern, J., Epstein, L. H. & Lerman, C. Using logistic regression to estimate delay-discounting functions. *Behav. Res. Methods Instrum. Comput.* **36**, 41–51 (2004).

11. Myerson, J., Baumann, A. A. & Green, L. Discounting of delayed rewards: (A)theoretical interpretation of the Kirby questionnaire. *Behav. Processes* **107**, 99–105 (2014).

12. Beran, M. J. *et al.* Chimpanzees (Pan troglodytes) can wait, when they choose to: a study with the hybrid delay task. *Anim. Cogn.* **17**, 197–205 (2014).

13. Beran, M. J. & Hopkins, W. D. Self-Control in Chimpanzees Relates to General Intelligence. *Curr. Biol.* **28**, 574-579.e3 (2018).

14. Paglieri, F. *et al.* The hybrid delay task: Can capuchin monkeys (Cebus apella) sustain a delay after an initial choice to do so? *Behav. Processes* **94**, 45–54 (2013).

15. Mies, G. W., Ma, I., de Water, E., Buitelaar, J. K. & Scheres, A. Waiting and working for rewards: Attention-Deficit/Hyperactivity Disorder is associated with steeper delay discounting linked to amygdala activation, but not with steeper effort discounting. *Cortex* **106**, 164–173 (2018).

16. Lejuez, C. W., Aklin, W. M., Zvolensky, M. J. & Pedulla, C. M. Evaluation of the Balloon Analogue Risk Task (BART) as a predictor of adolescent real-world risk-taking behaviours. *J. Adolesc.* **26**, 475–479 (2003).

17. Frey, R., Pedroni, A., Mata, R., Rieskamp, J. & Hertwig, R. Risk preference shares the psychometric structure of major psychological traits. *Sci. Adv.* **3**, e1701381 (2017).

18. Barron, G. & Erev, I. Small feedback-based decisions and their limited correspondence to description-based decisions. *J. Behav. Decis. Mak.* **16**, 215–233 (2003).

19. Hertwig, R., Barron, G., Weber, E. U. & Erev, I. Decisions from Experience and the Effect of Rare Events in Risky Choice. *Psychol. Sci.* **15**, 534–539 (2004).

20. Holt, C. A. & Laury, S. K. Risk Aversion and Incentive Effects. *Am. Econ. Rev.* **92**, 1644–1655 (2002).

21. Leiberg, S., Klimecki, O. & Singer, T. Short-Term Compassion Training Increases Prosocial Behavior in a Newly Developed Prosocial Game. *PLoS ONE* **6**, e17798 (2011).

22. Böckler, A., Tusche, A. & Singer, T. The Structure of Human Prosociality: Differentiating Altruistically Motivated, Norm Motivated, Strategically Motivated, and Self-Reported Prosocial Behavior. *Soc. Psychol. Personal. Sci.* **7**, 530–541 (2016).

23. Brohmer, H. *et al.* Inspired to Lend a Hand? Attempts to Elicit Prosocial Behavior Through Goal Contagion. *Front. Psychol.* **10**, 545 (2019).

24. Krockow, E. M., Colman, A. M. & Pulford, B. D. Cooperation in repeated interactions: A systematic review of Centipede game experiments, 1992–2016. *Eur. Rev. Soc. Psychol.* **27**, 231–282 (2016).

25. Krockow, E. M., Pulford, B. D. & Colman, A. M. Far but finite horizons promote cooperation in the Centipede game. *J. Econ. Psychol.* **67**, 191–199 (2018).

26. Rosenthal, R. W. Games of perfect information, predatory pricing and the chain-store paradox. *J. Econ. Theory* **25**, 92–100 (1981).

27. Berg, J., Dickhaut, J. & McCabe, K. Trust, Reciprocity, and Social History. *Games Econ. Behav.* **10**, 122–142 (1995).

28. Kreps, David M. Corporate culture and economic theory. in *Perspectives on positive political economy* 90–143 (Cambridge University Press, Cambridge, 1990).

29. Alós-Ferrer, C. & Farolfi, F. Trust Games and Beyond. *Front. Neurosci.* **13**, 887 (2019).

30. Johnson, K. L., Bixter, M. T. & Luhmann, C. C. Delay discounting and risky choice: Meta-analytic evidence regarding single-process theories. *Judgm. Decis. Mak.* **15**, 381–400.

31. Ashraf, N., Bohnet, I. & Piankov, N. Decomposing trust and trustworthiness. *Exp. Econ.* **9**, 193–208 (2006).

32. Cox, J. C. How to identify trust and reciprocity. *Games Econ. Behav.* **46**, 260–281 (2004).

33. Johnson, J. W. A Heuristic Method for Estimating the Relative Weight of Predictor Variables in Multiple Regression. *Multivar. Behav. Res.* **35**, 1–19 (2000).

34. Johnson, J. W. Factors Affecting Relative Weights: The Influence of Sampling and Measurement Error. *Organ. Res. Methods* **7**, 283–299 (2004).

35. Courville, T. & Thompson, B. Use of Structure Coefficients in Published Multiple Regression Articles: β is not Enough. *Educ. Psychol. Meas.* **61**, 229–248 (2001).

36. Lorenzo-Seva, U., Ferrando, P. J. & Chico, E. Two SPSS programs for interpreting multiple regression results. *Behav. Res. Methods* **42**, 29–35 (2010).

37. Liu, F. & Kong, Y. zoib: An R Package for Bayesian Inference for Beta Regression and Zero/One Inflated Beta Regression. *The R Journal* **7**, 34–51 (2015).

38. Bürkner, P.-C. **brms** : An *R* Package for Bayesian Multilevel Models Using *Stan*. *J. Stat. Softw.* **80**, (2017).

39. Nunnally, J. C. *Psychometric Theory (2nd Edition)*. (McGraw-Hill, New York, 1978).

40. Spearman, C. The Proof and Measurement of Association Between Two Things. in *Studies in individual differences: The search for intelligence.* (eds. Jenkins, J. J. & Paterson, D. G.) 45–58 (Appleton-Century-Crofts, East Norwalk, 1961). doi:10.1037/11491-005.

41. Hair, J. F., Black, Babin, B. J. & Anderson, R. E. *Multivariate Data Analysis*. (CENGAGE INDIA, Hamphire, U.;., 2018).

42. Zeller, R. A. & Carmines, E. G. *Measurement in the Social Sciences - The Link between Theory and Data*. (Cambridge University Press, Cambridge, 1980).

43. Reis, H. T. & Judd, C. M. *Handbook of Research Methods in Social and Personality Psychology*. (Cambridge University Press, Cambridge, 2014).

44. Cohen, J., Cohen, P., West, S. G. & Aiken. *Applied Multiple Regression/Correlation Analysis for the Behavioral Sciences*. (Routledge, 2013). doi:10.4324/9780203774441.
